# Optimization of a Soft Ensemble Vote Classifier for the Prediction of Chimeric Virus-Like Particle Solubility and Other Biophysical Properties

**DOI:** 10.3389/fbioe.2020.00881

**Published:** 2020-07-31

**Authors:** Philipp Vormittag, Thorsten Klamp, Jürgen Hubbuch

**Affiliations:** ^1^Institute of Engineering in Life Sciences, Section IV: Biomolecular Separation Engineering, Karlsruhe Institute of Technology, Karlsruhe, Germany; ^2^BioNTech SE, Mainz, Germany

**Keywords:** virus-like particles, solubility, hydrophobicity scales, machine learning, precipitation, optimization

## Abstract

Chimeric virus-like particles (cVLPs) are protein-based nanostructures applied as investigational vaccines against infectious diseases, cancer, and immunological disorders. Low solubility of cVLP vaccine candidates is a challenge that can prevent development of these very substances. Solubility of cVLPs is typically assessed empirically, leading to high time and material requirements. Prediction of cVLP solubility *in silico* can aid in reducing this effort. Protein aggregation by hydrophobic interaction is an important factor driving protein insolubility. In this article, a recently developed soft ensemble vote classifier (sEVC) for the prediction of cVLP solubility was used based on 91 literature amino acid hydrophobicity scales. Optimization algorithms were developed to boost model performance, and the model was redesigned as a regression tool for ammonium sulfate concentration required for cVLP precipitation. The present dataset consists of 568 cVLPs, created by insertion of 71 different peptide sequences using eight different insertion strategies. Two optimization algorithms were developed that (I) modified the sEVC with regard to systematic misclassification based on the different insertion strategies, and (II) modified the amino acid hydrophobicity scale tables to improve classification. The second algorithm was additionally used to synthesize scales from random vectors. Compared to the unmodified model, Matthew’s Correlation Coefficient (MCC), and accuracy of the test set predictions could be elevated from 0.63 and 0.81 to 0.77 and 0.88, respectively, for the best models. This improved performance compared to literature scales was suggested to be due to a decreased correlation between synthesized scales. In these, tryptophan was identified as the most hydrophobic amino acid, i.e., the amino acid most problematic for cVLP solubility, supported by previous literature findings. As a case study, the sEVC was redesigned as a regression tool and applied to determine ammonium sulfate concentrations for the precipitation of cVLPs. This was evaluated with a small dataset of ten cVLPs resulting in an *R*^2^ of 0.69. In summary, we propose optimization algorithms that improve sEVC model performance for the prediction of cVLP solubility, allow for the synthesis of amino acid scale tables, and further evaluate the sEVC as regression tool to predict cVLP-precipitating ammonium sulfate concentrations.

## Introduction

Protein solubility is a generally recognized problem in biopharmaceutical drug development. The fact that poor solubility can hamper a molecule’s development is a well-known challenge in chimeric virus-like particle (VLP) process development. VLPs are multimeric structures based on viral proteins, which are employed as vaccines, or delivery vehicles for proteins or nucleic acids (McAleer et al., 1984; Muratori et al., 2010; Kaczmarczyk et al., 2011; Strods et al., 2015; Bryan et al., 2016). For example, VLPs are applied as vaccines against hepatitis B virus or human papillomavirus (McAleer et al., 1984; Bryan et al., 2016). Chimeric VLPs (cVLPs) are VLPs decorated with foreign epitopes altering the function of the unmodified VLPs by, for example, directing the patient’s immune response toward the inserted epitope (Yoshikawa et al., 1993; Klamp et al., 2011). While this flexibility of antigenic display is one of the major advantages of VLPs (Pumpens et al., 2008), recombinant insertion of epitopes often results in expression of insoluble structures (Karpenko et al., 2000; Billaud et al., 2005). Factors affecting cVLP solubility have been described as, for example, insert charge (Whitacre et al., 2009), amino acid side chain volume (Karpenko et al., 2000), or the content of specific amino acids, such as tryptophan or arginine (Vormittag et al., 2020). None of these individual attributes describe the cVLP solubility landscape comprehensively. This is underlined by findings, in which combining different attributes improved the solubility model’s performance (Vormittag et al., 2020). Each amino acid makes a unique contribution to protein solubility, e.g., based on its charge, volume, or specific interactions. In recent years, a great number of so-called hydrophobicity scales have been derived that aim to serve in an (almost) calibration-free model to describe hydrophobicity-related problems based on amino acid-specific hydrophobicity values.

Already in 1962, Tanford pointed out that hydrophobic interaction is a key factor influencing the stability of globular protein conformation (Tanford, 1962). Nozaki and Tanford (1971) measured transfer free energies of amino acid side chains into ethanol and dioxane, deriving an early hydrophobicity scale. They describe the hydrophobicity scale value of an amino acid as, for example, its tendency to be located in the interior of a protein. This idea of a scale to describe an amino acid’s tendency to partition into exterior or interior regions of a protein is an assumption that does not take into account 3-D-specific effects. If 3-D-specific effects were negligible, a linear or non-linear function should exist that perfectly describes a protein’s solubility based on its amino acid composition. The fact that this is probably not the case has been extensively shown, directly or indirectly, for example, by several studies on protein solubility prediction yielding only about 60–80% accuracy (Idicula-Thomas et al., 2006; Smialowski et al., 2006; Magnan et al., 2009; Hebditch et al., 2017), or detailed mechanistic studies on protein structure and assembly. The latter is illustrated by the complex behavior of VLPs. Tyrosine can be regarded as a hydrophobic (aromatic ring) or polar (hydroxyl group) amino acid. Interestingly, it is required for Hepatitis B core antigen (HBcAg) to form capsids, buried in a hydrophobic pocket (Wynne et al., 1999). A mutational form, replacing tyrosine 132 by alanine, prohibits particle assembly (Bourne et al., 2009). The predominant quaternary structure of this HBcAg mutant is therefore a dimer instead of the 180- or 240-meric capsid. This comes with great changes in physicochemical and biophysical behavior as the mass of a solvatized entity differs by 90- to 120-fold. Obviously, this behavior cannot be explained by one universal hydrophobicity scale, as this is an effect with a strong 3-D spatial component.

In a recent article by our group, we applied a soft ensemble vote classifier (sEVC) with embedded feature selection to predict cVLP solubility, based on 91 hydrophobicity scales (Vormittag et al., 2020) to harness the information contained in multiple scales. This can help overcome the limitations of a sequence-based approach by expanding the dimensionality of the sequence-based descriptions by using different scales in one model. In said study, a feature selection algorithm selected the best features to be included in the model based on a training set. Individual hydrophobicity scale performance for classification ranged from 54 to 85%, which underpins that hydrophobicity scales cannot be universal. The choice of hydrophobicity scales by the algorithm and the analysis of the best- and worst-performing scales revealed dominant roles for arginine and tryptophan in cVLP solubility. In another study on the prediction of peptide aggregation propensity, feature selection has been successfully employed to select the best of 560 features, showing some overlap with regard to best features with our previous study (Fang et al., 2013). Both these publications combine theoretical physicochemical data with statistical methods to predict a biophysical property by selecting appropriate physicochemical measures. Compared to pure statistical regression, these models therefore contain physicochemical information, which is advantageous for calibration on smaller datasets and for interpretation of the data.

Zviling et al. (2005) came to similar conclusions in their work on the prediction of transmembrane helical regions. Based on two existing amino acid scales, provided by Kyte and Doolittle and Goldman, Engelmann and Steitz (Kyte and Doolittle, 1982; Engelman et al., 1986), they generated a set of new hydrophobicity scales by optimization using a genetic algorithm on a cross-validation set. Both Zviling’s and our approach combine real experimental physicochemical data, contained in hydrophobicity scales, with a statistical adjustment to the problem to be modeled. This ensures that prediction is based on actual physicochemical groundwork. The degree of statistical adjustment, however, is larger, when a 20-dimensional function is optimized, such as by optimization of scale tables in Zviling’s work, than with calibration of decision trees that only shift classification borders in the one-dimensional target function space.

The present article describes approaches to optimize and tweak our recently developed model to improve prediction accuracy, learn more about the data, and to extend the model to other biophysical parameters. An optimization procedure for the synthesis of amino acid scale tables is one approach to improve model performance. To ensure that overfitting is avoided, this approach would benefit from a large balanced dataset, as was used in our recent study. These synthesized scales would be tailored to the problem they are optimized on and therefore have the potential to improve model performance and reveal dominant roles of amino acids for classification of the dataset.

In our previous study, we demonstrated the potential of optimizing the model’s prediction based on the contingency matrices of the individual insertion strategies (Vormittag et al., 2020). The dataset used consisted of 568 chimeric HBcAg constructs, created by a grid of 71 different inserts and eight insertion strategies. The eight different insertion strategies in this study define where in the major immunodominant region of the HBcAg molecule the epitope is inserted and which amino acids are deleted. The different strategies are meant to optimize the integration of the foreign epitope into the VLP sequence and would ideally result in an integration that produces a soluble cVLP. Analyzing the strategies showed that the model systematically overestimated or underestimated certain insertion strategies with respect to the predicted solubility (Vormittag et al., 2020). To recapitulate briefly, a strategy that is overrepresented in the training false-positive (*FP*) group has overestimated solubility in relation to the other strategies. This means that this strategy is particularly bad for solubility in the perspective of the training dataset. The model is, at this stage, incapable of describing this different behavior. As previously suggested, this could be related to 3-D phenomena that cannot be described by a sequence-based approach (Vormittag et al., 2020). Knowledge of the above-described systematic misclassification helps (a) to conclude that this insertion strategy may be disadvantageous with respect to solubility, and (b) to adjust the model, so that the model’s blind spot is compensated. The latter can be achieved by modifying the model predictions specifically for those insertion strategies, for which systematic misclassification can be observed in the training set.

The introduction of a foreign epitope to be displayed on the VLP surface has implications on many facets of the product and the process. The main question addressed by our work – the solubility of cVLP candidates after cell lysis – is typically a decision point where candidates drop out of the candidate pool. In this large dataset, this leaves half of the candidates to choose from Vormittag et al. (2020). This number will be cut down to very few candidates throughout the development process. Besides solubility, several other biophysical, or physicochemical parameters are determinants in the development process of a cVLP candidate. The most important property is the candidates’ ability to induce an immune response against the target structure, the basis for its efficacy (Klamp et al., 2011; Roseman et al., 2012; Frietze et al., 2016). Therefore, the introduced foreign epitope has to be properly displayed and accessible on the molecular surface, which is something that can very probably not be described by amino acid scale-based models and requires detailed 3-D structural studies (Roseman et al., 2012). Another process-related property that can vary among the candidates is their structural and phase behavior as a function of the solution environment. VLPs are complex nanostructures which are held together by intra- and intermolecular forces, such as electrostatic and hydrophobic interactions and disulfide bonds. Their complex structural behavior is dependent on the introduced foreign epitope. In a previous work by our group, we investigated the re-assembly of disassembled HBcAg cVLPs (in the form of HBcAg dimers) by increasing ionic strength by diafiltration (Rüdt et al., 2019). We observed that the diafiltration volumes – an indicator of progress in buffer exchange and therefore ionic strength – that were required to complete the VLP assembly reaction varied between the three constructs. Based on zeta potential measurements, this behavior could be related to surface charge. In another study, a high-throughput 3-D structure generation workflow was developed that we applied on exactly these three constructs in their disassembled form to calculate a surface charge that correlated well with the zeta potential measurements (Klijn et al., 2019). This is a good example of *in silico* representations of physicochemical properties, which pave the way for model-assisted rather than empirical process development. This said, it seemed promising to test the sEVC model to predict other process-related properties. One such property is the required concentration of ammonium sulfate to precipitate cVLPs, a typical process step in cVLP downstream processing (Hillebrandt et al., 2020). Precipitation of cVLPs can typically be achieved with an ammonium sulfate concentration that leaves most of the contaminants in solution (Kazaks et al., 2017). Once the supernatant containing these contaminants is discarded, the cVLPs can be resolubilized, resulting in high yields with the potential of increasing product concentration. The ammonium sulfate concentration required for cVLP precipitation is typically determined in screening experiments (Hillebrandt et al., 2020). To reduce required time and resources, regression for the estimation of the ammonium sulfate concentration for different cVLPs would therefore be highly interesting.

We have recently shown that ensembles of individual classifiers based on hydrophobicity scales and amino acid sequences are potent classifiers for cVLP solubility. The objective of this study is to evaluate the potential of different optimization strategies to improve our recently developed sEVC framework and to apply the sEVC to another biophysical parameter. We therefore combined the sEVC with optimization algorithms to improve generated models and to learn more about the data obtained. Optimization algorithms employed in this study aimed to (I) reduce systematic misclassification based on insertion strategies, (II) optimize and generate amino acid scale tables, and (III) combine both optimization strategies to maximize model performance. Finally, we show some perspective on how to apply the model to another biophysical parameter, i.e., ammonium sulfate concentration for cVLP precipitation, by transforming the model to a regression tool.

## Materials and Methods

### Dataset

For an overview of the methodology applied to this work, we recommend reading our previous article on the sEVC for chimeric VLP solubility prediction (Vormittag et al., 2020). The dataset is equivalent to that used in said previous study, comprising amino acid sequence and binary solubility data of chimeric HBcAg constructs. Chimeric HBcAg was based on C-terminally truncated, His-tagged Hepatitis B virus core protein, modified with 71 different inserts and eight unique insertion strategies. An insertion strategy describes where in the major immunodominant region of HBcAg the foreign epitope is inserted and how many amino acids of the native protein are deleted. All possible combinations of the 71 inserts and eight strategies result in 568 constructs/observations. The literature hydrophobicity scales used in this study can be found in our recent work and the Supplementary Table S1, originally derived from AAindex (Kawashima et al., 2007), the SPLIT 4.0 server (Juretić et al., 1993), and ProtScale (Gasteiger et al., 2005), and put together by Simm et al. (2016). Reversed scales were treated as duplicates, and therefore removed if a non-reversed scale was available, resulting in 91 hydrophobicity scales. For all models, a training set of 384 observations was used, which was created once by stratified sampling based on the identity of the inserts and insertion strategies (Vormittag et al., 2020). The remaining 184 observations were used as an external test set. For Monte Carlo cross-validation (MC-CV), a 1:1 random split of the training set was applied to each validation run.

### Soft Ensemble Vote Classifier

The sEVC applied in this study is described in detail in the above-mentioned recent study by our group and was only slightly modified. Briefly, the sEVC aggregates the solubility predictions of individual classifiers, which classify based on hydrophobicity features calculated by hydrophobicity scales and sequence data. The features *F*_*p*_ are derived by accumulating hydrophobicity values of amino acids *Hyd*(*aa*), as prescribed by a hydrophobicity scale, for the entire amino acid sequence [*aa*_1_, *aa*_2_, …, *aa*_*n*_] of each observation *p* Eq. (1).

(1) $$F_{p}=\sum_{aa_{1,p}}^{aa_{n,p}}Hyd\left(aa_{i,p}\right)$$

Classifiers are one-level decision trees induced from these hydrophobicity features, trained using Gini’s diversity index as impurity measure (Gini, 1912; Windeatt and Ardeshir, 2004). The classifiers return a class (“soluble”/+1 or “insoluble”/-1) with a probability associated with the respective child node in the decision tree. The classifier’s vote *v* is the probability with the sign associated with the respective class and therefore falls between −1 and +1. Aggregation of all votes *v*_*i*_ results in the continuous prediction value *p* of the sEVC, which is normalized by the number of included scales *n*, again falling between −1 and +1, as explained by Eq. (2).

(2) $$p=\frac{\sum_{i=1}^{n}v_{i}}{n}$$

This continuous prediction value is subsequently discretized, where for *p* > 0, the prediction is “soluble” or +1 and for *p* ≤ 0, it is “insoluble” or −1. In the sEVC, an embedded feature selection algorithm informs about the potency of the individual classifiers to predict solubility and sorts them according to their feature importance, namely their Matthew’s correlation coefficient (MCC) on prediction of the training data as defined in Eq. (3).

(3) $$MCC=\frac{TP\times TN-FP\times FN}{\sqrt{\left(TP+FP\right)\left(TP+FN\right)\left(TN+FP\right)\left(TN+FN\right)}}$$

*TP*, *TN*, *FP*, and *FN* are true-positive, true-negative, false-positive, and false-negative classifications (contingency matrix of training, validation, and test set). In our previous study, feature selection was based on accuracy, which is, however, biased when unbalanced datasets are considered (Powers, 2011). For model validation, an MC-CV procedure with embedded feature selection is run to inform about the optimal number of included classifiers. The sEVC could theoretically be composed of any combination of available classifiers, where each combination is an individual model. In this study, the *n* best classifiers, according to feature selection, are included, where *n* ranges from 1 to the maximum number of available classifiers. For the dataset of literature scales, *n* is 91. In the scale generation procedure described below, *n* ranges from 1 to 16.

The training/validation set is selected randomly from the full training set in each MC-CV run. The sEVC including 1 to *n* classifiers, sorted by descending feature importance, is probed on these MC-CV datasets. In the original study on the sEVC, model validation datasets were newly constructed for each of the 91 models, while in this study, the same MC-CV dataset within one MC-CV run is used for all *n* models. This is reasonable as each of the models is evaluated on the same dataset within one MC-CV run, thus increasing comparability while reducing computational resources. The validation procedure informs about the model performance dependent on the number of included classifiers. This information can be used for generation of the final model based on all training data to predict the external test set.

### Optimization Based on Insertion Strategies

**FIGURE 1:**
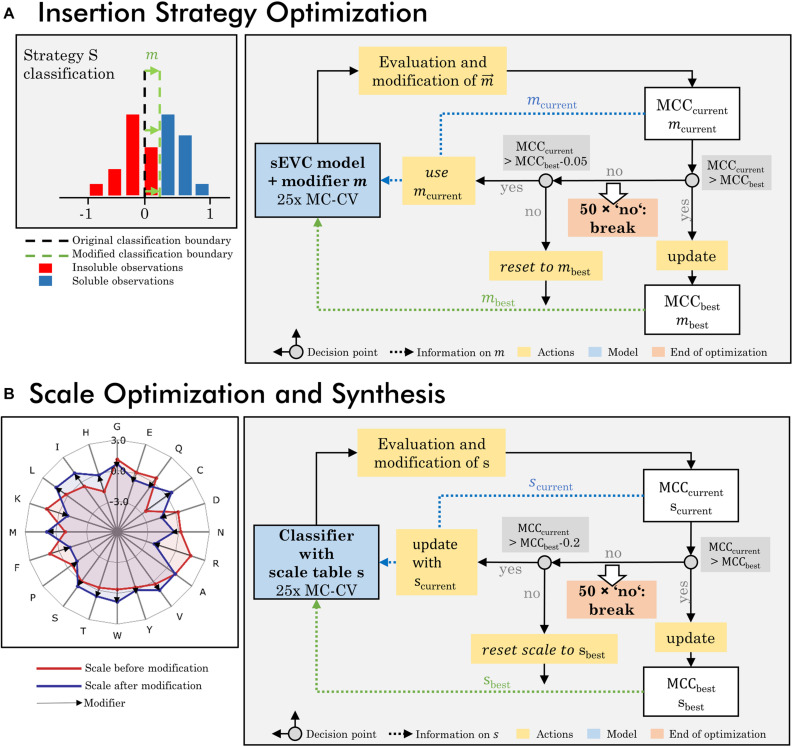
Workflow of optimization procedures. **(A)** The insertion strategy optimization is based on modifying the classifier for a specific strategy to increase model performance. In a 25-fold Monte Carlo cross-validation (MC-CV), the previous modifier is evaluated and a new modifier is derived, based on systematic misclassification in the false-positive and negative group, specific for certain insertion strategies. This results in the current modifier *m*_current_ and current MCC_current_. If MCC_current_ is better than previous best MCC, the best MCC and modifier are updated and used for the next iteration. If the MCC_current_ is lower than the best MCC within a defined acceptance margin of 0.05, the current modifier is used for the next iteration. If it is below this acceptance margin, the MCC and modifier are reset to the previous best MCC and modifier. If for 50 times, no improvement on the MCC has been made, the iteration is stopped. **(B)** Scale optimization and synthesis are based on an optimization of the individual amino acid’s hydrophobicity values in the hydrophobicity scale. In each iteration the previous scale is modified and probed in 25-fold MC-CV, resulting in a current MCC and scale *s*. The iteration rules are comparable to the insertion strategy optimization, with the difference that the acceptance margin is higher with 0.2 and the modified scales, as opposed to the modifiers in the insertion strategy optimization, are stored and updated.

An optimization algorithm focusing on the insertion strategies was developed, based on a loop of model generation, evaluation, and modification (Figure 1A). The dataset used for this optimization procedure is the training set of 384 observations and all 91 literature hydrophobicity scales (Supplementary Table S1). In the first iteration, a 25-fold MC-CV is computed and an accumulated validation set contingency matrix is calculated. This matrix contains information on the accumulated number of validation *TP*, *TN*, *FP*, and *FN* classifications dependent on the insertion strategy for all 25 MC-CV runs. The largest absolute value of *FN-FP* classifications defines which strategy’s prediction is modified in this iteration and what the sign of this modification is. If *FN* > *FP*, it is positive, if *FN* < *FP*, it is negative. A strategy that has more *FN* than *FP* should be classified more positively by the classifier, in order to push *FN* observations into the *TP* group. This is realized by modifying the accumulated continuous prediction values [compare also Eq. (2)] of the sEVC. A modification vector *m* contains the information on how this aggregated prediction value is modified individually for each strategy, imaginable as shifting the classification boundary (Figure 1A; while, in fact, the predictions are shifted instead of the classification boundary). In each iteration, the vector is changed by an absolute 0.01 for the strategy and sign identified as described above. The first iteration is calculated on an unmodified model, providing the modification vector for the second iteration.

In each iteration, the sEVC modified by the previous modification vector is evaluated in the 25-fold MC-CV, resulting in a new median validation MCC value, i.e., target function to be maximized. This value is the median of all models’ median validation MCC. Therefore, the target function takes into account the entire model space of 1 to 91 included classifiers. If this is in an acceptable range (equal to or at maximum 0.05 worse than the best previous median MCC), then the accumulated contingency matrix of this iteration is used to calculate a new modification vector for the next iteration. The acceptable range was determined in pre-experiments, so that early termination of the algorithm was avoided while limiting model deterioration. If the MCC is better than the best MCC so far, the modification vector is stored as best modification vector, and future iterations are compared to this MCC. If the MCC is worse and outside the acceptable range, the modification vector is reset to the current best modification vector. If for 50 times, no improvement on the MCC has been made, the optimization is stopped. For the evaluation of optimized model performance, the model with the best MCC during optimization is chosen and the respective modification vector is applied within the sEVC in order to predict in 1000-fold MC-CV and to predict the external test set. For each of the 91 models, the validation procedure results in a median validation MCC and accuracy. For evaluation, the median of these values (“overall median”) is compared for the MCC and accuracy, respectively. The metric to compare initial and optimized model performance is the change of these values in percent, where the difference between optimized and initial performance values is divided by their maximum range, i.e., 1 and 2 for accuracy and MCC, respectively.

### Synthesis of Amino Acid Scales

A second algorithm was created to modify amino acid scales to (I) synthesize new amino acid scales and (II) optimize existing scales specifically for the presented VLP solubility problem (Figure 1B). The two algorithms are almost identical and are, in the following, explained by the example of scale synthesis. Each scale is optimized from an initial scale that contains normally distributed pseudorandom numbers for each of the 20 encoded amino acids. In each iteration, the scale *s* of the preceding run is adjusted with a modifier *m* Eqs (8, 9). The modifier is designed to move the average *FP* and *FP* feature value in the direction of the classification boundary, which is the cut point of the one-level decision tree, thus aiming to decrease false classification. This is done independent of insertion strategies. The modifier’s direction is determined by average feature values of the classification groups *FN* and *FP* and the difference in frequency of individual amino acids. The average feature values *F*_*FN*_ and *F*_*FP*_ and mean amino acid frequency vectors *a*_*FN*_ and *a*_*FP*_ are derived from a 25-fold MC-CV run with the scale *s* of the previous iteration. Herein, the amino acid frequency vectors describe the frequency of the individual amino acids within the groups of FN and FP classification, respectively. The average feature values *f*_*FN*_,*f*_*FP*_, and their differences are

(4) $$f_{FN}=a_{FN}^{\prime }s,$$

(5) $$f_{FP}=a_{FP}^{\prime }s,$$

and

(6) $$\Delta f=f_{FN}-f_{FP}.$$

The vector of the difference in amino acid frequency is given by

(7) $$\Delta a_{FN,FP}=\left(a_{FN}-a_{FP}\right)$$

The modifier used in this optimization loop is vector

(8) $$m=\Delta f\Delta a_{FN,FP},$$

which is used in a centered and unit variance-scaled form $\bar{m}$. In each iteration, a scale is modified as prescribed in Eq. (9).

(9) $$s_{i+1}=s_{i}+r\bar{m},$$

where *s*_*i*_ and *s*_*i*+1_ are the previous and the modified scale, respectively, and *r* is the modification rate, which was 1% for scale synthesis. After modification, the new scale is also centered and scaled to unit variance. Therefore, the extent of modification is comparable in all iterations, as it corresponds to an average of 1% of unit variance.

The modified scale is probed in a 100-fold MC-CV run resulting in a median MCC value. This current MCC value is compared to the best previous MCC value. If it is better, it is stored as the new best MCC value with associated new best scale. If it is worse, the new scale is still accepted, as long as the MCC does not fall below an acceptance margin, which is 0.2, where the scale and MCC are reset to the previous best iteration. The acceptance margin is larger than in the insertion strategy-based optimization, as model performance fluctuated more with this second optimization strategy. If no new best scale is created for 50 times consecutively, the optimization is stopped and the best scale and MCC are returned. For the generation of scales, either the full training set or subsets thereof were used. When the full training set is used, one scale is generated by the algorithm. When two (equally sized) subsets are used, two scales are generated by the algorithm. The algorithm was run with up to 16 subsets, which in turn resulted in 16 different scales. Subsets were either created by random split into evenly sized subsets or split by insertion strategies.

Additionally, this algorithm was used to optimize literature scales. Based on the full training set with 384 observations, the 91 literature scales were used as scales in an initial iteration, where the optimization was performed for each scale individually at a rate of 5%. Other parameters were identical to the scale generation procedure.

### Analysis of Performance Data for Optimizations

Evaluation of optimized and non-optimized models was always based on 1000-fold MC-CV, returning median MCC and median absolute deviation (MAD) of MCC. The external test set consisted of 184 observations, remaining after stratified sampling of 384 training observations from the full dataset. For all models, the same external test set was used.

### Redesigning the Model for Regression of Precipitation Data

Ten cVLP constructs of strategy H were experimentally evaluated for cVLP-precipitating ammonium sulfate concentration. The ammonium sulfate concentration screening procedure was performed as described in a recent article on precipitation of HBcAg VLPs (Hillebrandt et al., 2020). Briefly, clarified *Escherichia coli* lysate, containing HBcAg VLPs, was adjusted to 0.25% polysorbate 20 and then precipitated with 4 M ammonium sulfate stock solution to different target concentrations. The ammonium sulfate concentration required to precipitate most of the cVLPs was determined visually based on SDS PAGE scans.

Scales generated by the above-described algorithm, that is, those derived from randomly splitting the training set into eight equal parts, were used to train a model based on all observations with insertion strategy H. Evaluation of the model was performed on the prediction of the 1000-fold MC-CV set for eight models composed of 1–8 classifiers. As opposed to the classification for solubility, the continuous prediction value of the models [compare also Eq. (2)] was not discretized. The mean resulting prediction value of the MC-CV runs was subsequently used to be correlated with the experimental data in linear regression. The order of the scales was derived from feature selection. The data were fit using MATLAB’s *fitlm* function and evaluated by the ordinary *R*^2^.

## Results and Discussion

### Optimization Based on Insertion Strategies

An optimization procedure was developed, which, based on 25-fold MC-CV, adjusts the model’s predictions for the insertion strategies individually based on a modification vector. This modification vector is applied before discretizing the continuous scale of the aggregated sEVC votes by increasing (higher solubility) or decreasing (lower solubility) the continuous prediction value.

**FIGURE 2:**
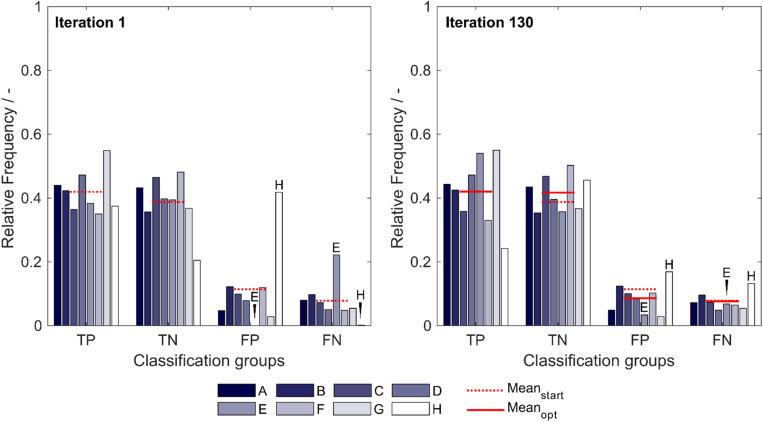
Relative frequency of classification groups based on insertion strategies **(A–H)** in the first iteration (left) and the best optimization iteration (right) during insertion strategy-based optimization with the 91 literature scales. The mean of the relative frequencies within a classification group is shown for the first iteration (Mean_start_) and for the best optimization (Mean_opt_), indicating that through optimization, the *FP* group decreases in mean relative frequency while the TN group increases in mean relative frequency. Strategies E and H are marked additionally to guide the eye. TP: true positive; TN: true negative; FP: false positive; and FN: false negative.

The optimized model, obtained after 130 iterations, showed an increase in median validation MCC values from 0.63 to 0.69 (Table 1). Most notable modifications are made on predictions of insertion strategies E and H, resulting in a strategy-specific accuracy increase of 12% for both strategies, while the MCC increased by 8% for strategy E and decreased by 1% for strategy H. This is also illustrated by the number decrease of these strategies in the respective false classification groups (Figure 2). Overall, there is a similar true-positive (*TP*), and false-negative (*FN*) number, indicated by the mean over insertion strategies (red line), while true negative (*TN*) is increased and false positive (*FP*) decreased. The constant numbers in *FN* are explained by the increase in the number of strategy H in this group, balancing out the decrease of strategy E in *FN*. Making strategy H more negative pushed *FP*-classified observations to *TN*, but also *TP* to *FN*. Strategy E performed better in this regard, as we only see a minor increase in the number of E in *FP*.

**TABLE 1:** Modification vector and summarized model Monte Carlo cross-validation (MC-CV) performance data for the insertion strategy optimization.

Best modification vector for insertion strategy-based optimization only									
Strategy	A	B	C	D	E	F	G	H	
*m*	0.01	0	−0.01	0	0.59	−0.1	0	−0.5	

**Insertion strategy-based optimization only**									

**Strategy**	**A**	**B**	**C**	**D**	**E**	**F**	**H**	**I**	**Overall median**

A_start_	0.87	0.78	0.83	0.87	0.78	0.83	0.92	0.58	0.81
A_opt_	0.88	0.78	0.83	0.87	0.90	0.83	0.92	0.70	0.84
A_change_	1%	0%	0%	0%	12%	0%	0%	12%	4%
MCC_start_	0.75	0.56	0.66	0.74	0.64	0.67	0.83	0.39	0.63
MCC_opt_	0.76	0.56	0.65	0.74	0.79	0.66	0.83	0.37	0.69
MCC_change_	1%	0%	0%	0%	8%	0%	0%	–1%	3%

**Insertion strategy-based optimization combined with scale generation and optimization**									

Optimization of literature scales:							A_opt_		0.86
							A_change*_		5%
							MCC_opt_		0.72
							MCC_change*_		5%
Generation of scales: Example subset S_8,1_:							A_opt_		0.86
							A_change*_		5%
							MCC_opt_		0.73
							MCC_change*_		5%

**FIGURE 3:**
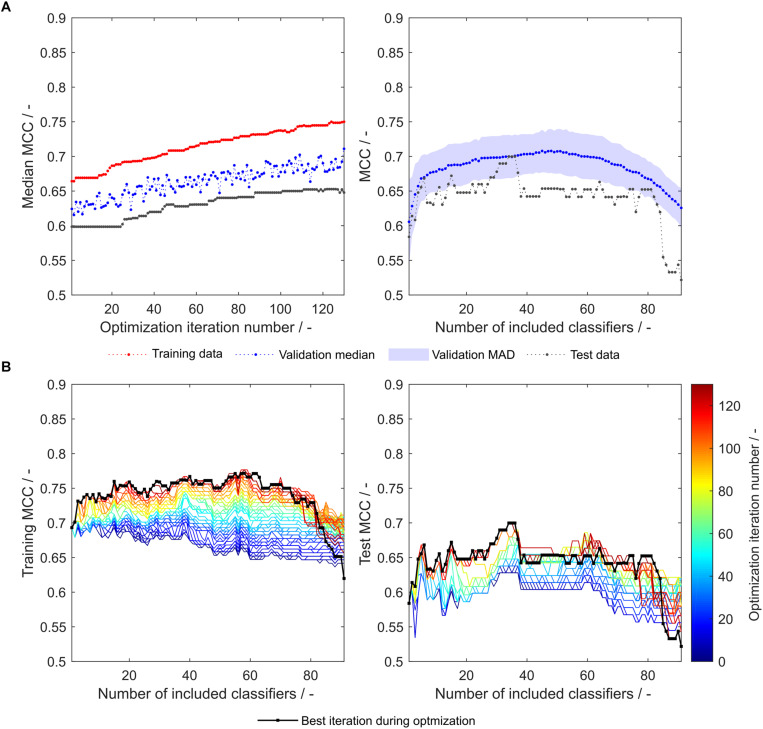
Matthew’s correlation coefficient (MCC) during insertion strategy-based model optimization. Scales used were the unmodified 91 literature scales. Median MCC are shown for training, validation, and test data over optimization iterations (**A**, left). Validation and test MCC are shown over number of included classifiers in the soft ensemble vote classifier (sEVC; **A**, right) for the best model in the optimization procedure. The median absolute deviation (MAD) of the validation MCC above and below the median validation MCC is visualized with a shaded area. Training and test MCC over number of included classifiers are shown for the optimization iterations until the best iteration, where median validation MCC was highest **(B)**. Optimization iterations are illustrated by a colormap, where dark blue represents the first iteration and dark red the best iteration, highlighted by the black dots.

The final modification vector (m) with elements for each strategy is shown, affecting the continuous prediction values after aggregation of all votes (Figure 1A). Accuracy (A) and Matthew’s Correlation Coefficient (MCC) before (start) and after optimization (opt) are shown for each strategy. The percent change values in accuracy and MCC are calculated by the absolute change in accuracy and MCC, respectively, divided by the range of the statistical value (1 for accuracy, 2 for MCC). Overall median is based on the median performance data of the 1000-fold MC-CV. The MC-CV results in 91 values, which are the median of the 1000 MC-CV runs for each number of included classifiers individually. These values are illustrated for the optimized model in Figure 3A, right. The overall median describes the median of these 91 values, which is the optimization target function. Overall median MCC and accuracy are also shown for strategy-based optimization of scales optimized and synthesized with the scale table optimization algorithm. *Change of MCC and accuracy of optimized/generated scales are calculated relative to the performance of literature scales without optimization (A_start_ and MCC_start_).

During the optimization, both median validation and training MCC as well as external test set MCC of 91 models are increasing (Figure 3A, left). Their maxima approximately coincide, underpinning the usefulness of the validation MCC-based optimization procedure. While this median MCC of all models (including 1–91 classifiers) describes the general tendency of model improvement, it is also valuable to have a closer look on the improvement of the individual 91 models. During the optimization, both training and test MCC increase for most models, when 1 to about 80 classifiers are included (Figure 3B). However, models deteriorate at roughly > 80 included classifiers. This said, the most important area is where the MCC is maximal (30–40 for the test set). Here, the optimization algorithm continuously improves the models with regard to training and test set MCC, where the last iteration shows highest MCC values for the individual models. To select the appropriate number of included classifiers, validation data is useful. The validation data of the optimized model generally follows the course of the external test data (Figure 3A, right). Their maxima do not coincide. However, choice of the best model with regard to validation MCC also produces a reasonable model for the prediction of test data with a test MCC of 0.65 at 48 included scales. Interestingly, the optimum number of included classifiers with regard to test MCC is 34 with an MCC of 0.70 (Table 2), similar to an optimum of 29–30 included classifiers as described in our previous study with the basic sEVC (Vormittag et al., 2020).

**TABLE 2:** Performance data of selected models on the external test set.

	91 LS	91 LS,SO	91 LS_opt_, SO	S_1,5_	S_5,17_	S_8,1_	S_8,1,_SO
MCC_max_	0.63	0.70	0.72	0.77	0.77	0.72	0.76
MCC_vali,max_	0.63	0.65	0.71	0.77	0.75	0.72	0.71
A_vali,max_*	0.81	0.83	0.85	0.86	0.88	0.86	0.85

*Accuracy is shown for the model with best validation MCC. Best models’ performance data are written in bold. LS, Literature scales; SO, Strategy optimization; LS_opt_, Scales optimized with scale table optimization workflow; S_x,y_, Generated scale table with x scales, optimization procedure y of 20. Best test set Matthew’s Correlation Coefficient (MCC) and test MCC of model with best validation MCC in 1000-fold MC-CV are shown.

### Synthesis and Optimization of Amino Acid Scale Tables

Another option to optimize the model relates to the amino acid scale tables. The target for such optimizations was seen in the feature values and amino acid composition in the *FP* and *FN* groups. Adaptation of the scale tables was performed, in a way that amino acids predominant in the respective groups were altered in their scale table values to push observations that have been predicted falsely over the classification boundary, i.e., decision tree cut point. This is illustrated by the following example. Let us assume that *FP* has a lower mean feature value than *FN*, and, for example, that valine has a higher frequency in *FP* than *FN*. Observations in *FP* are classified positive but their data label is negative or insoluble. If we wanted observations of *FP* to be classified rather insoluble, their feature value would have to be increased for false observations to cross the classification boundary. This said, the classification boundary is not static, but changes with alterations in the amino acid scale table. Therefore, small increments are made and scale improvement is monitored. Note that the aim is to increase *FP* hydrophobicity, but decrease *FN* hydrophobicity. Considering that valine is more frequently observed in the *FP* group, increasing valine’s hydrophobicity value in the scale would be beneficial, as it would increase the average *FP* hydrophobicity value more than the average *FN* hydrophobicity value. If this is executed for all amino acids, the *FP* feature value would ideally be increased and the *FN* feature value decreased, increasing overall correlation.

**FIGURE 4:**
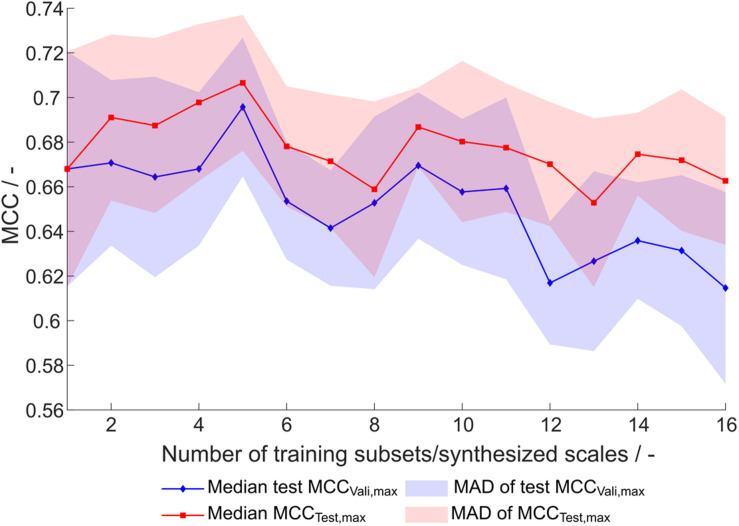
Test set Matthew’s correlation coefficient (MCC) of synthesized scales. For each number of training subsets/synthesized scales, 20 repetitions of scale generation were performed. Median and median absolute deviation (MAD) of best test set MCC and test set MCC of best model by validation MCC are shown.

This optimization procedure has been performed on the entire training dataset (384 observations) and equally sized subsets, where the number of subsets, and therefore synthesized scales, was 1–16, resulting in subsets with 384 to 24 training examples. Each of the optimization procedures was performed 20 times, resulting in 320 scale tables S_[number of scales], [number of repetition]_. For evaluation of the synthesized scales, the MCC of the external test set prediction at optimal number of classifiers as determined by validation (maximum validation MCC) is compared (Figure 4). This validation-based model selection is a useful strategy to select the optimal number of included classifiers and thus the model. Additionally, the maximum MCC of the external test set prediction is evaluated. Both metrics increase to a maximum from one to five generated scales, where the median of maximum test MCC is 0.71 and the median of test MCC at maximum validation MCC is 0.70. From this maximum toward a higher number of training subsets and likewise number of synthesized scales, there is a tendency of decreased model performance. Also, best test MCC and test MCC at best validation diverge more, probably since training subsets become smaller and the probability of more unrepresentative scales being synthesized rises, thus potentially decreasing the power of validation for model selection. From this data and with the present dataset, it would be recommended to synthesize scales from five subsets, although most other scales also perform reasonably well.

The overall best scale table with regard to best test MCC at maximum validation MCC is S_1,5_ with test MCC_vali,max_ = 0.77 (Table 3), which is significantly better than with literature scale tables with test MCC_vali,max_ = 0.63. With respect to the 20 repetitions of scale synthesis, median test MCC at maximum validation MCC for one scale is worse than for five scales (Figure 4). The best scale table with five scales, S_5,17_, shows MCC_vali,max_ = 0.75. For the best model by validation MCC, these scale tables show a test set accuracy of 0.86 and 0.88, respectively, corresponding to 155 and 158 correctly classified constructs in the test set of 184 observations.

**TABLE 3:** Best five scale tables, measured by highest test set Matthew’s Correlation Coefficient (MCC) at maximum validation MCC.

	1 Scale		3 Scales						5 Scales				
	S_1,5_	S_1,20_	S_3,3_			S_3,16_			S_5,17_				
				
MCC_vali,max_/Amino acid	0.77	0.76	0.76			0.76			0.75				
A	–0.320	0.092	1.087	–0.088	–0.281	1.371	–0.161	–0.056	0.847	–0.143	0.493	0.784	0.418
R	0.168	0.312	0.454	0.867	0.201	–0.122	–0.460	0.194	–1.902	–0.845	–1.492	0.044	–0.306
N	–0.668	–0.017	0.046	1.390	0.217	–0.403	–0.519	–1.132	0.050	0.377	–1.687	1.458	–1.545
D	–0.751	–1.758	–0.771	–2.164	–0.555	–0.817	–0.375	–1.697	0.159	0.165	–0.368	–0.836	0.724
C	0.182	1.395	0.801	–0.660	2.344	2.143	–1.505	–1.437	–0.719	0.893	0.549	0.205	0.302
Q	0.410	0.256	–0.263	0.240	0.009	–0.898	0.285	0.181	0.015	0.386	0.913	0.047	–0.444
E	–0.345	–0.656	–0.283	–0.942	–0.318	–0.396	–0.198	–0.298	–0.439	–0.444	–0.984	–0.744	–0.262
G	0.422	0.284	–0.289	–0.563	–0.190	–1.103	–1.659	–1.955	–0.073	1.105	1.775	2.545	0.446
H	–0.552	–0.659	–2.698	0.134	–0.891	–2.272	0.337	0.884	1.179	–1.168	–1.083	–0.052	–1.557
I	–0.321	0.795	0.271	0.059	–0.316	0.446	–0.310	–0.338	0.982	0.223	0.128	–1.003	–0.436
L	0.090	–0.118	–0.008	0.298	–0.478	–0.010	–0.276	0.371	–0.789	–1.022	0.432	–0.893	–0.393
K	0.383	0.269	–0.286	–0.014	0.018	–0.266	0.216	0.034	–1.569	1.379	–0.576	–0.050	0.505
M	–0.730	–0.445	–0.601	–0.904	–2.212	–0.072	–1.271	–0.301	1.773	–2.126	–0.550	–1.816	1.239
F	3.038	–0.027	0.944	0.552	–0.294	1.117	0.822	1.835	–0.206	–0.410	0.055	–0.426	2.380
P	0.747	1.745	–0.576	1.395	0.980	0.227	0.395	1.206	0.751	0.865	–0.661	0.760	1.058
S	0.645	0.077	–1.055	0.426	0.385	–0.687	0.475	0.367	–0.062	–0.027	1.154	–0.702	–0.276
T	–1.853	–2.579	–0.003	–1.159	–0.334	–0.107	1.314	1.178	1.089	–1.397	0.735	–0.759	–0.249
W	0.604	1.002	2.331	1.474	2.216	1.450	2.898	0.673	0.224	1.988	0.980	0.854	–0.010
Y	–1.469	–0.766	0.743	–1.394	–0.766	–0.022	–0.204	–0.498	–1.803	0.030	–1.130	–0.281	–1.918
V	0.320	0.797	0.157	1.054	0.267	0.421	0.195	0.788	0.491	0.171	1.317	0.863	0.326

*Scale tables are centered and scaled to unit variance.*

The generation of subsets for scale synthesis was additionally investigated with subsets containing one insertion strategy each, amounting to eight different subsets. The median of maximum test set MCC and test MCC at maximum validation MCC were 0.67 ± 0.03 and 0.65 ± 0.03, respectively (data not shown). They were comparable to randomized training subset generation with eight subsets, showing a maximum test set MCC and a test MCC at maximum validation MCC of 0.66 ± 0.04 and 0.65 ± 0.04, respectively, (Figure 4). Therefore, strategy-based generation of subsets for scale synthesis was not advantageous to random subset generation.

**FIGURE 5:**
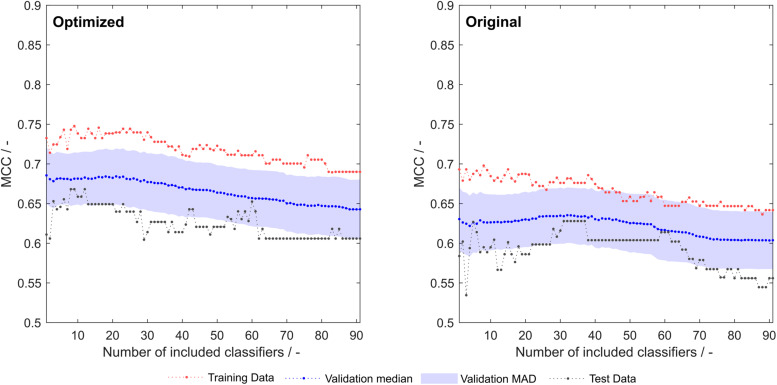
Training, validation and test sets Matthew’s correlation coefficient (MCC) of optimized **(left)** and original **(right)** 91 literature hydrophobicity scales. The shaded area represents the median absolute deviation (MAD) during 1000-fold Monte Carlo cross-validation.

Optimization of the 91 literature scales with the same algorithm that synthesized scales as described above resulted in an improvement of training, validation and test set MCC over the whole model space (Figure 5). A greater rate during optimization was chosen (5%), as the lower rate employed for scale synthesis resulted in early optimization termination with no significant improvements in model performance (data not shown).

### Combination of the Optimization Procedures

**FIGURE 6:**
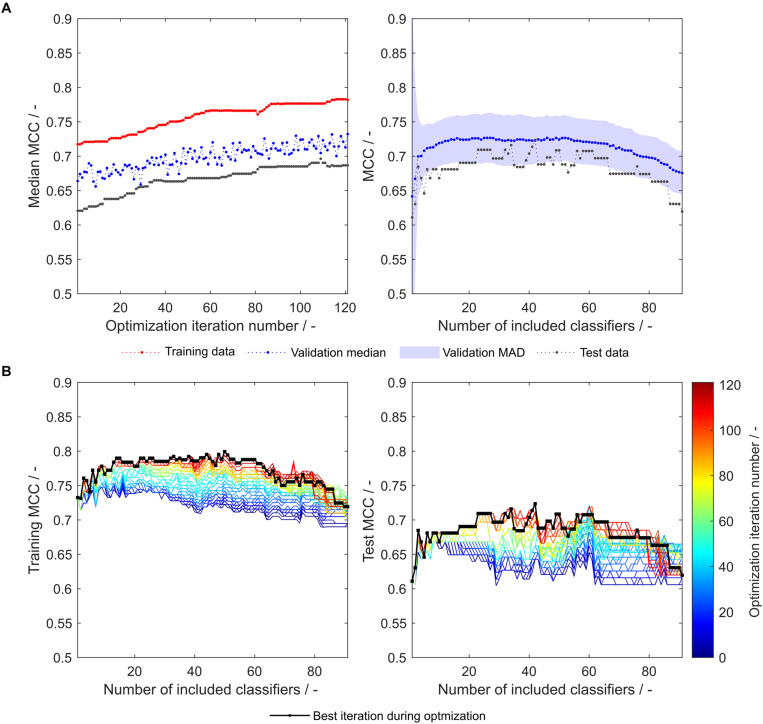
Matthew’s correlation coefficient (MCC) during insertion strategy-based model optimization. Scales used were the optimized 91 literature scales (see also Figure 4). Median MCC are shown for training, validation, and test data over optimization iterations (**A**, left). Validation and test MCC are shown over number of included classifiers in the soft ensemble vote classifier (sEVC; **A**, right) for the best model in the optimization procedure. The median absolute deviation (MAD) of the validation MCC above and below the median validation MCC is visualized by a shaded area. Training and test MCC over number of included classifiers are shown for the optimization iterations until the best iteration, where median validation MCC was highest **(B)**. Optimization iterations are illustrated by a colormap, where dark blue represents the first iteration and dark red the best iteration, highlighted by the black dots.

As both above-described optimization procedures tackle different challenges, it seems promising to combine these by adding an insertion strategy-based optimization procedure after synthesizing or optimizing hydrophobicity scales. Strategy-based optimization of optimized literature scale tables results in similar trends but increased model performance compared to models with unmodified literature scale tables (compare Figures 3, 6). With optimized scale tables, the resulting MCC values are higher for the training, validation and test sets (Figure 6A). The maximum test set MCC and the test set MCC at maximum validation MCC are increased to 0.72 and 0.71, respectively, as compared to 0.70 and 0.65 before scale table optimization (test set performance data summarized in Table 2). Additionally, the performance of the model at very low and high numbers of included classifiers is benefitted, never falling below an MCC of 0.6 for the test set (Figure 6B). The number of insertion strategies in the false classification groups show similar trends as without scale table optimization, underlining that the insertion strategy-based optimization procedure is effective (Supplementary Figure S1).

**FIGURE 7:**
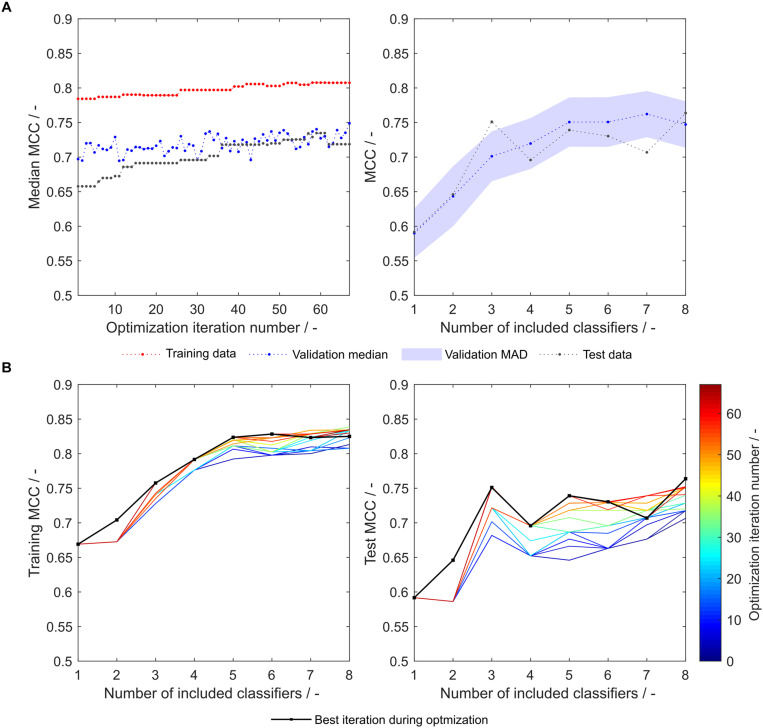
Matthew’s correlation coefficient (MCC) during insertion strategy-based model optimization. Scales used were eight generated scales from scale table set S_8,1_. Median MCC are shown for training, validation, and test data over optimization iterations (**A**, left). Validation and test MCC are shown over number of included classifiers in the soft ensemble vote classifier (sEVC; **A**, right) for the best model in the optimization procedure. The median absolute deviation (MAD) of the validation MCC above and below the median validation MCC is visualized by a shaded area. Training and test MCC over number of included classifiers are shown for the optimization iterations until the best iteration, where median validation MCC was highest **(B)**. Optimization iterations are illustrated by a colormap, where dark blue represents the first iteration and dark red the best iteration, highlighted by the black dots.

As another example, the first set of scales generated from eight training subsets (S_8,1_) was tested with the strategy-based optimization algorithm. Figures 7A,B show that synthesized scales still can benefit from this optimization procedure resulting in higher MCC values for training, validation and test sets for most models. Compared to the 91 literature scales, these models perform 5% better with regard to validation accuracy and MCC (Table 1). Model test set MCC is increased for all models except for the sEVC including seven scales, which remains at a comparable test set MCC as before strategy-based optimization (Figure 7B), resulting in slightly decreased test MCC and accuracy at best validation MCC (Table 2). This shows that an improvement in median performance of models does not necessarily result in an improved prediction outcome. Additionally, well-performing scale tables such as S_1,5_ and S_5,17_ were tested with the strategy-optimization workflow. However, strategy-based optimization failed to improve model performance using these scales, suggesting that with these scales, systematic misclassification based on insertion strategies is not an issue. This in turn shows, that this systematic misclassification can be reduced by the use of other scale tables, and not only by the strategy-based optimization. This contradicts the assumption that insertion strategy-based misclassification is a 3-D-specific effect that cannot be captured by an amino acid sequence-based approach (Vormittag et al., 2020). The strategy-based optimization could theoretically be performed for all 20 × 16 generated scale sets, but would go beyond the scope of this research.

### Correlation of Scales Within Scale Tables

**FIGURE 8:**
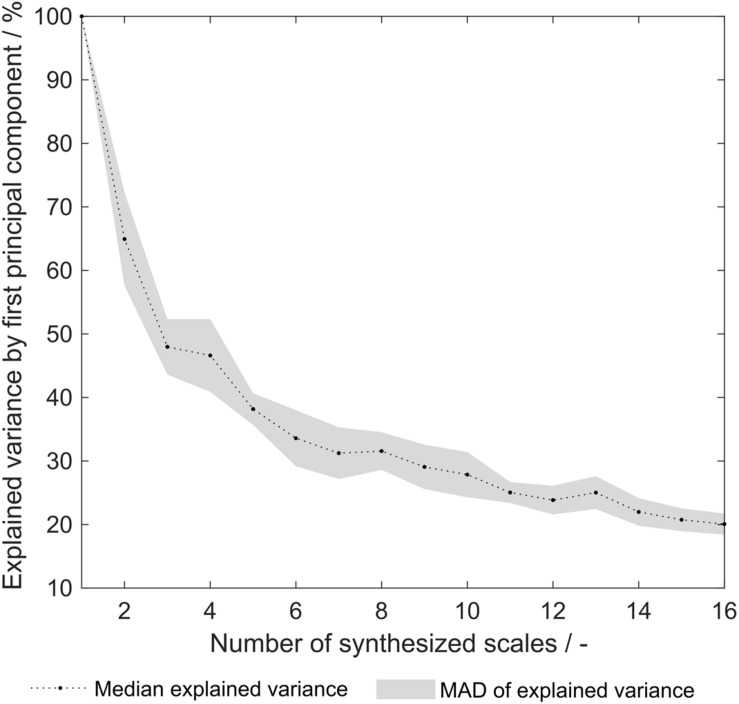
Explained variance by first principal component of synthesized hydrophobicity scale tables. For each number of synthesized scales, corresponding to the number of training subsets that were generated for scale synthesis, the median of 20 repetitions is shown. The shaded area represents the median absolute deviation (MAD).

As pointed out earlier, the explained variance of the first principal component (PC) from a principal component analysis (PCA) on the 91 literature scales revealed that already 69% of the variance is explained with one single PC (Vormittag et al., 2020). This indicates that a significant degree of correlation between the 91 literature scales is present. After the optimization procedure, this explained variance remained at a comparable level of 66% (data not shown). The explained variance of the synthesized scale tables’ first PC after PCA varied from 100 to 20% (Figure 8). An explained variance of 100% is predefined for the situation where only one scale was generated from the training set, as the first PC equals this scale. From 2 to 16 scales, the explained variance is below the above- mentioned value for the literature scales. It can therefore be deduced that the correlation between synthesized scales is reduced as compared to literature scales. This can be interpreted as increased orthogonality, which was expected to increase model performance of the sEVC. Decreased correlation between scales could explain the improved performance of synthesized amino acid scales in the ensemble of classifiers, as described above. PCA of the group of scales synthesized from dataset division by the eight insertion strategies reveals that with 28.3 ± 3.3% of explained variance, this approach is comparable to random division into eight insertion strategies with 31.6 ± 3.0% (data not shown). This suggests that the correlation between generated scales can probably not be reduced by splitting the dataset by the insertion strategies. As discussed above, model performance did not improve with this subset generation strategy either.

### Amino Acids With Characteristic Hydrophobicities

**FIGURE 9:**
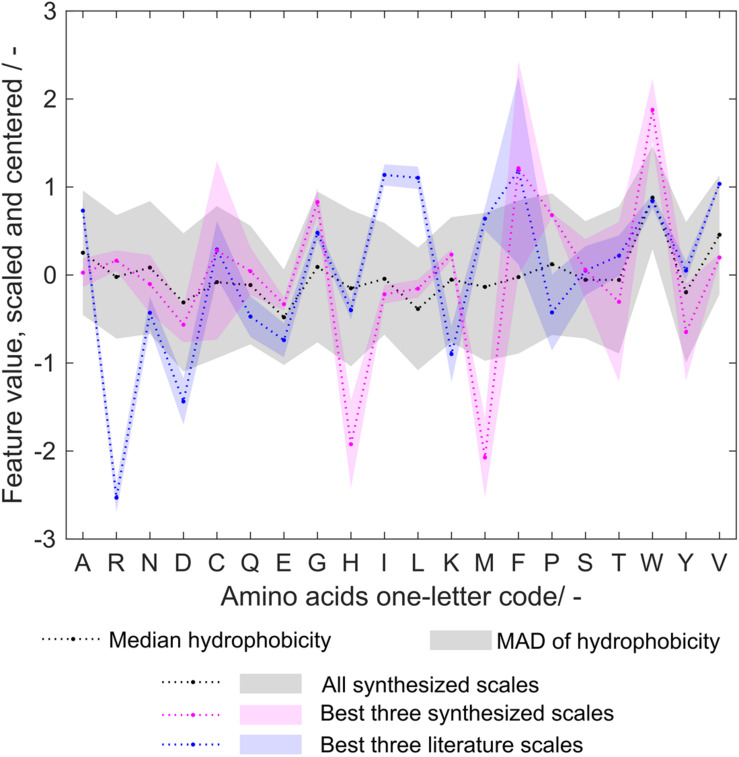
Median and median absolute deviation (MAD) of amino acid hydrophobicity for all synthesized, the three best synthesized, and the three best literature scales. The hydrophobicity scales are centered and scaled to unit variance. For comparison purposes, the sign of hydrophobicity scales was changed so that tryptophan (W) hydrophobicity was always positive. Amino acids are represented with one-letter code. The MAD is visualized by a shaded area.

The three best literature scales by feature selection in 1000-fold MC-CV show very similar hydrophobicity values (Figure 9). This is partly the case because they are either related to each other or because they were generated from similar original scales (von Heijne and Blomberg, 1979; Eisenberg et al., 1982; Zviling et al., 2005). All synthesized scales taken together show a rather broad distribution around zero, with few more prominent exceptions, such as valine (V), or tryptophan (W). Interestingly, the three best synthesized scales also seem to agree quite well on most of the amino acids’ hydrophobicities, which are, however, for many examples different from the best literature scales (Best 25 individual scales shown in Supplementary Table S2). The largest difference can be seen in the arginine (*R*) hydrophobicity values. The literature scales’ low arginine value, indicating lowest hydrophobicity, make them exceptional with respect to worse-performing literature scales, suggesting an important role of arginine for VLP assembly and solubility (Vormittag et al., 2020). This is not confirmed with the synthesized amino acid scales. This being said, it is also not contradicted. To be able to interpret what the synthesized amino acid scale tables mean, one has to consider the mechanism behind the optimization algorithm. In the algorithm, the scales are analyzed for misclassification, and the resulting feature values of misclassified observations. On the basis of the amino acid frequency distribution within the classification groups, the hydrophobicity scale is optimized, thus fitting the scale to the training data of 384 observations through the MC-CV-based procedure. Therefore, the synthesized scales can be regarded as hydrophobicity scales that describe the cVLP solubility problem well. Their application to other molecules or biophysical data would yet have to be probed. (A small case study regarding other biophysical data is shown below.) The discrepancy between the hydrophobicity values, for example for arginine, is probably due to the dominance of other amino acids with respect to their influence during the optimization procedure. This underpins the usefulness of approaching the solubility problem both from a physicochemical and statistical perspective. Tryptophan plays a very important role being one of the most hydrophobic amino acids in the synthesized scales, while its hydrophobicity is less pronounced for literature scales. Its high hydrophobicity value contributes to insoluble classification. In accordance with this finding, amino acids with large side chains, such as tryptophan, have been described to be problematic for HBcAg cVLP assembly (Karpenko et al., 2000).

Methionine (M) and histidine (H) show low hydrophobicity values in the best three synthesized scales, but have a median hydrophobicity close to zero considering all scales. A one-level decision tree based on histidine content was constructed on the entire dataset and showed a low MCC of 0.17 (data not shown), indicating that its low hydrophobicity might be an artifact of the random scale initiation along with its irrelevance to classify the observations. A decision tree on methionine resulted in an MCC of 0.41. However, observations with large methionine content would be rather classified insoluble with this decision tree. This speaks for a high hydrophobicity, as opposed to what can be seen for the three best synthesized scales.

In summary, model performance was significantly enhanced by the synthesis of scales. The above- described cases yet underline the potential to further optimize the procedure for scale synthesis. However, when scales are increasingly optimized, it is important to bear in mind the danger of overfitting.

### Redesigning the Soft Ensemble Vote Classifier for Estimation of Ammonium Sulfate Concentrations for VLP Precipitation

Apart from cVLP solubility, there is a variety of other biophysical properties that are interesting with regard to cVLP processing. In a previous study, we investigated precipitation and redissolution of a cVLP candidate (Hillebrandt et al., 2020). In this work, ammonium sulfate concentration to precipitate the cVLP is determined in a screening experiment before running the process. The screening method to determine optimal ammonium sulfate concentrations for precipitation of the cVLPs was applied to ten cVLPs, all constructed with insertion strategy H, contained in the present dataset. As an example model, synthesized scales from eight training subsets were fitted to solubility data of all observations with insertion strategy H. Synthesized scales were used instead of literature scales, as these were generated based on the model space of interest. Eight models were created including 1–8 of the scales sorted by feature importance. Instead of discretizing the prediction of the models, their continuous value was retrieved. Thus, the individual classifiers become regression models. However, we will still call them “classifiers” in this section for consistency. In principle, this continuous prediction value should be positive for all constructs as they had to be soluble to be investigated experimentally for precipitation behavior. The rationale behind using the continuous value is that constructs for which the classifier is uncertain have biophysical properties that are actually close to insolubility and therefore probably easier to precipitate.

**FIGURE 10:**
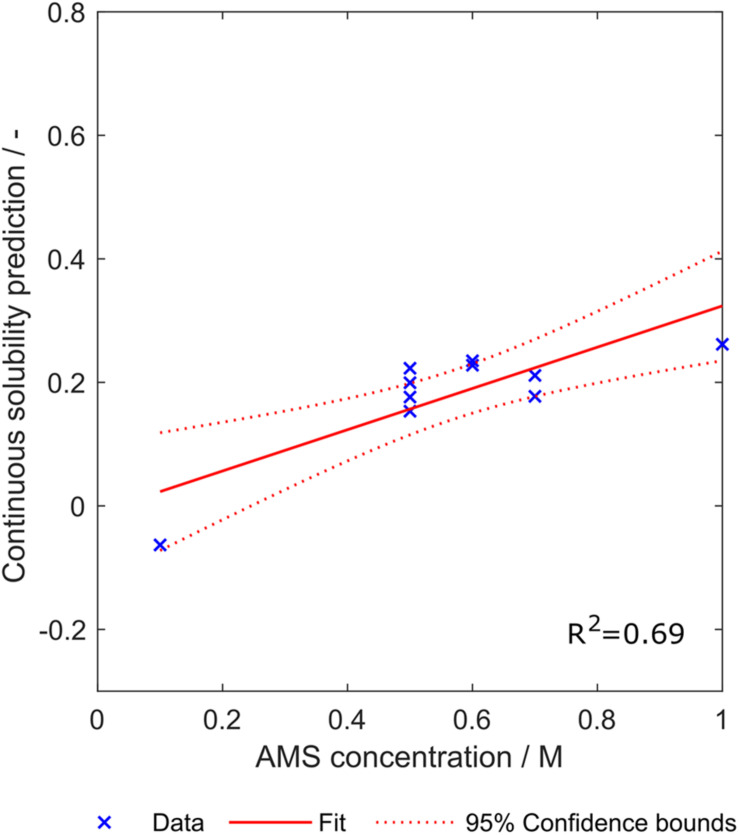
Relationship between the continuous solubility prediction value and optimal ammonium sulfate concentration for precipitation of ten constructs. Eight scales were used, which were generated with a scale table optimization procedure (Set S_8,1_). Goodness of fit is indicated by 95% confidence bounds and *R*^2^.

The ammonium sulfate concentration required to precipitate the investigated ten constructs was mostly between 0.5 and 0.7 M, except one concentration with 0.1 M and another concentration with 1 M ammonium sulfate (SDS PAGE scans not shown). Linear regression with an sEVC based on scales from set S_8,1_ including all eight synthesized scales resulted in an ordinary *R*^2^ of 0.69. This indicates a linear correlation between the continuous solubility prediction and the ammonium sulfate concentration required for precipitation (Figure 10). Confidence bounds are wider at the edge data points of 0.1 M and 1 M ammonium sulfate. This is due to a higher data density in the middle region. The linear fit almost crosses the *y*-axis at 0 M ammonium sulfate concentration, which, as discussed above, reflects a behavior of this model that would be expected. The construct with lowest continuous solubility prediction value precipitates at low ammonium sulfate concentrations of only 0.1 M. Interestingly, it would be classified as insoluble by the algorithm, while in fact being a soluble construct. Its closeness to the solubility classification border is probably the reason for the low associated precipitating ammonium sulfate concentration.

It is important to note that the dataset of ammonium sulfate concentrations is comparably small. This regression study therefore serves as a proof-of-concept, demanding a larger dataset for confirmation of the results and for refinement of the method. With the limited amount of data available, it cannot be deduced which number of included classifiers is optimal for the regression model. While for this set of scales S_8,1_, it seems that increasing classifier numbers boost regression performance (see also Supplementary Figure S3), the use of other scales shows inverse trends, where using the first (and according to feature selection best) classifier results in the best *R*^2^, e.g., set S_9,1_ (data not shown). This indicates that regression for the estimation of required ammonium sulfate concentration for precipitation of cVLPs would benefit from a validation procedure, realizable with larger datasets. Additionally, the relationship between the continuous prediction value and ammonium sulfate concentration was assumed to be linear, due to the limited data available. However, this might also be inappropriate, which again could be answered with a larger dataset. Not all 16 × 20 scale tables have been tested, since it was deemed inappropriate given the small dataset. Finding the right set of scales by testing all 320 scale table sets for 10 experimental data points can quickly lead to overfitting. The first scale table of the set with eight scales has been chosen, as it represents an average number of generated scales. From some additional tests with other scale tables, it might be assumed that a small number of generated scales perform worse than a greater number (data not shown), which would have to be confirmed with a larger dataset of ammonium sulfate concentration data.

## Conclusion and Outlook

In this study, we have developed and evaluated two different optimization algorithms to improve the performance of an sEVC for the prediction of cVLP solubility based on amino acid sequences and hydrophobicity scale tables. The dataset in this study consisted of 568 chimeric HBcAg constructs, created by insertion of 71 different foreign peptide sequences using 8 different insertion strategies. The sEVC algorithm was originally developed to classify based on 91 literature hydrophobicity scales but showed systematic misclassification for some of the insertion strategies. This was tackled by optimizing the prediction specific for these insertion strategies, resulting in a strategy-specific increase in validation accuracy and MCC of up to 12 and 8%, respectively. The second optimization algorithm modified amino acid scale tables and was also used to synthesize 320 different hydrophobicity scale table sets showing an MCC and accuracy of up to 0.77 and 0.88, respectively, on the external test set of 184 HBcAg constructs. The presented models are therefore better than other protein solubility models, typically reporting accuracies of about 0.60 to 0.80. A combination of both procedures could elevate the prediction performance data of worse-performing synthesized scales to similar levels. Finally, extension of the model to regression of the required ammonium sulfate concentration for precipitation of ten cVLPs was evaluated, and the linear correlation showed a promising *R*^2^ of 0.69. The results of this study encourage to further explore the model for other biophysical parameters and molecules.

## Data Availability Statement

The raw data supporting the conclusions of this article will be made available by the authors, without undue reservation, to any qualified researcher. The amino acid sequence data for this article cannot be made available because they are confidential industry data.

## Author Contributions

JH initiated and supervised the work and edited the manuscript. TK provided the solubility data and was involved in the generation of the idea behind this manuscript. PV evolved the solubility prediction approach, created the ammonium sulfate concentration data, developed the optimization algorithms presented in this manuscript, performed the computational work and statistical analysis, and drafted the manuscript. PV, TK, and JH read and approved the final manuscript. All authors contributed to the article and approved the submitted version.

## Conflict of Interest

TK was employed by the company BioNTech SE. The remaining authors declare that the research was conducted in the absence of any commercial or financial relationships that could be construed as a potential conflict of interest.

## Abbreviations

cVLP: chimeric virus-like particle
FN: false negative
FP: false positive
HBcAg: hepatitis B virus core antigen
MAD: median absolute deviation
MCC: Matthew’s correlation coefficient
MC-CV: Monte Carlo cross-validation
PC: principal component
PCA: principal component analysis
sEVC: soft ensemble vote classifier
TF: true false
TP: true positive
VLP: virus-like particle.
