# Supplementary material for: Optimization of a Soft Ensemble Vote Classifier for the Prediction of Chimeric Virus-Like Particle Solubility and Other Biophysical Properties
Source: Front Bioeng Biotechnol. 2020 Jul 31;8:881. doi: 10.3389/fbioe.2020.00881 (PMC7411134; doi:10.3389/fbioe.2020.00881)
Supplement: Supplementary file 1 [file Data_Sheet_1.PDF]

Supplementary Material

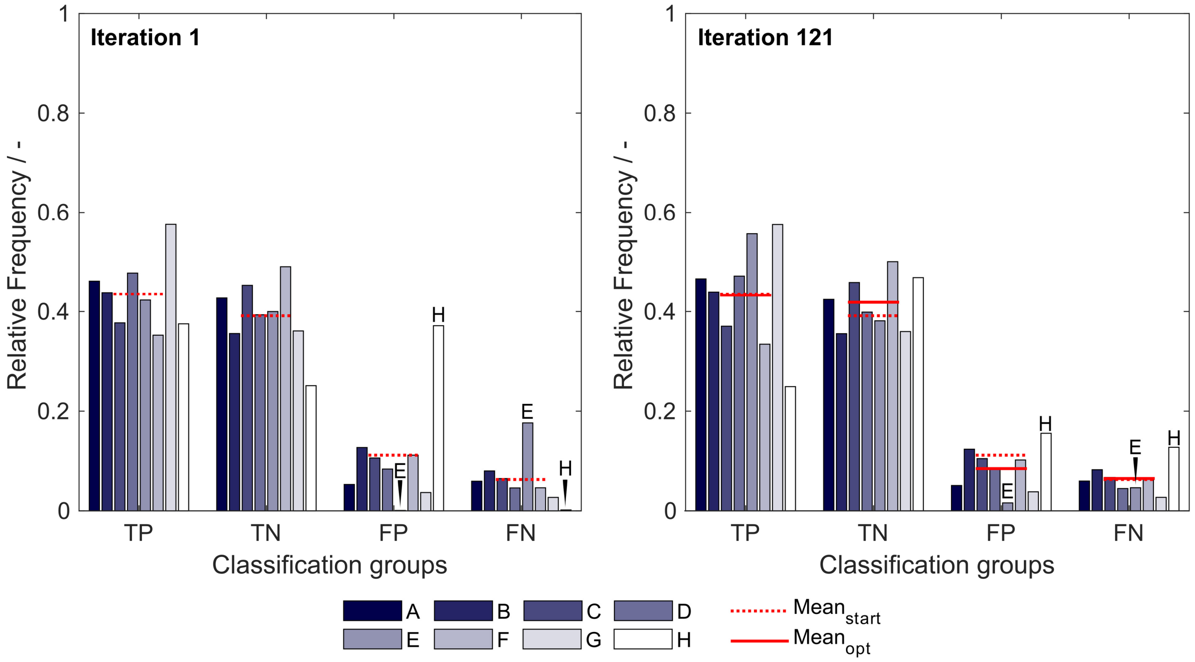

Figure S1: Relative frequency of classification groups based on insertion strategies A-H in the first iteration (left) and the best optimization iteration (right) during insertion strategy-based optimization with the 91 optimized literature scales. The mean of the relative frequencies within a classification group is shown for the first iteration (Mean$_{start}$) and for the best optimization (Mean$_{opt}$), indicating that through optimization the FP group decreases in mean relative frequency while the TN group increases in mean relative frequency. Strategy E and H are marked additionally to guide the eye. TP: true positive; TN: true negative; FP: false positive; FN: false negative.

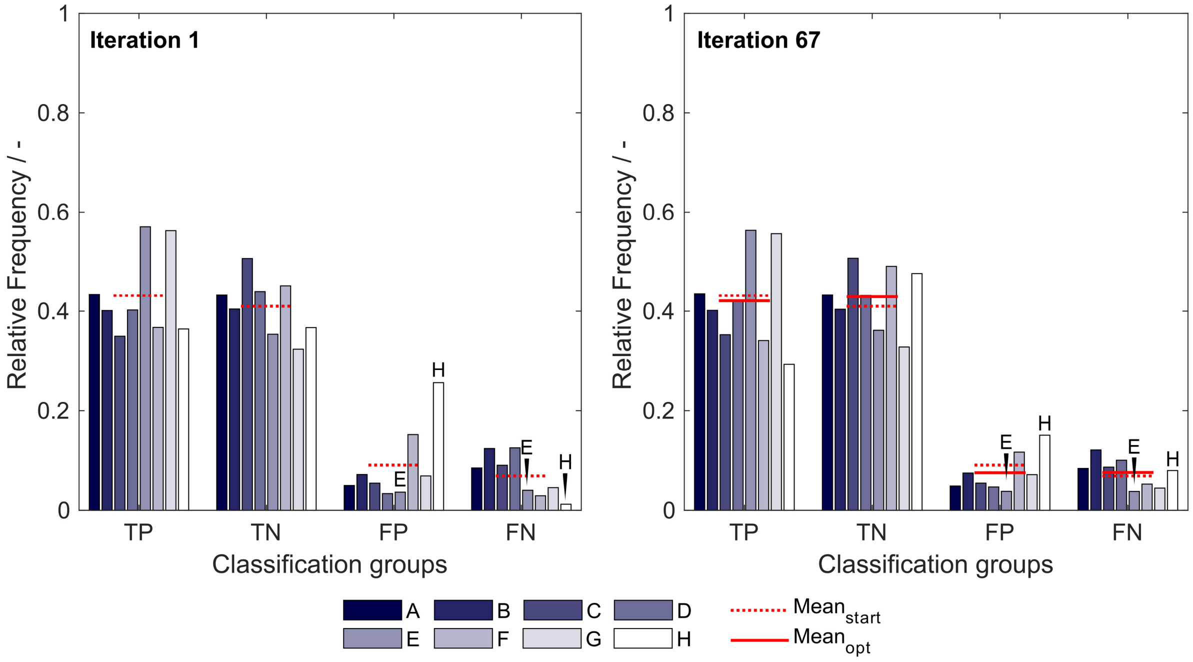

Figure S2: Relative frequency of classification groups based on insertion strategies A-H in the first iteration (left) and the best optimization iteration (right) during insertion strategy-based optimization with the synthesized scale set $S_{8,1}$. The mean of the relative frequencies within a classification group is shown for the first iteration (Mean$_{start}$) and for the best optimization (Mean$_{opt}$), indicating that through optimization the FP group decreases in mean relative frequency while the TN group increases in mean relative frequency. Strategy E and H are marked additionally to guide the eye. TP: true positive; TN: true negative; FP: false positive; FN: false negative.

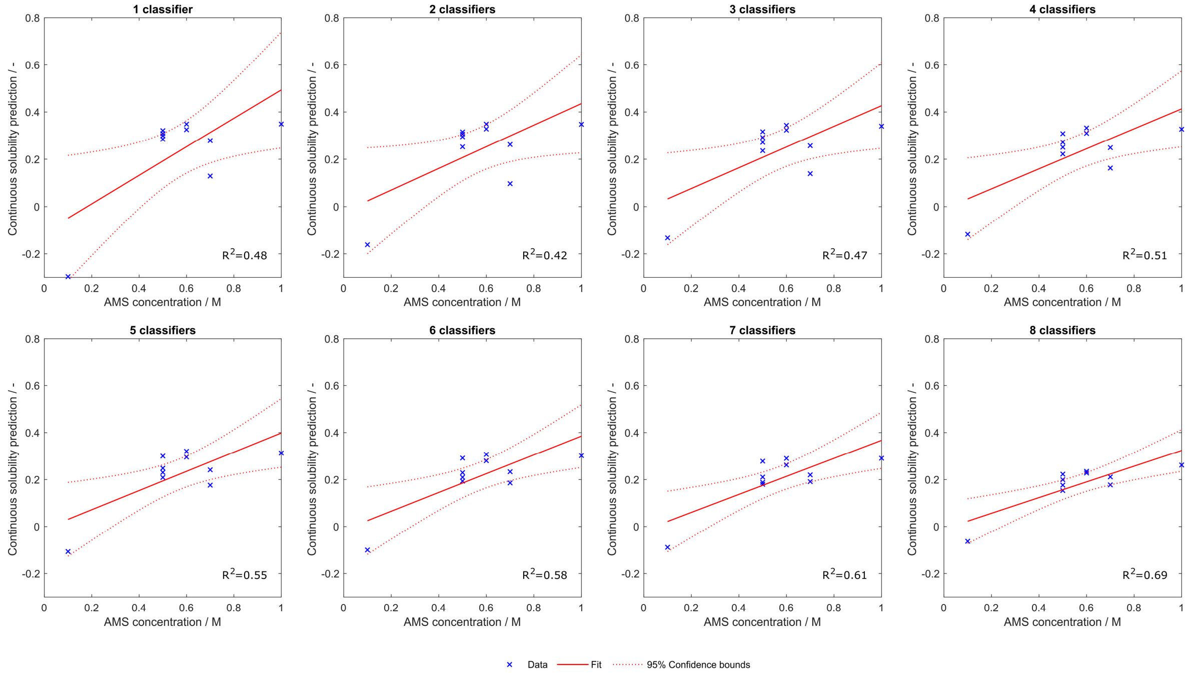

Figure S3: Relationship between continuous solubility prediction value and optimal ammonium sulfate concentration for precipitation of ten constructs. In eight models, 1-8 scales were used, which were generated with a scale table optimization procedure (Set $S_{8,1}$). 95% confidence bounds and R^2 indicate goodness of fit.

Table S1: Centered and unit-variance scaled literature hydrophobicity scales derived from Simm *et al.* (2016). For original references of the scales, the reader is referred to the publication of Simm *et al.* (2016). Reversed scales were excluded if there was a complementary, non-reversed scale available, resulting in 91 scales. Each amino acid, represented in single-letter code, is assigned a hydrophobicity value by each hydrophobicity scale.

**SCALE-ID**/AMINO ACID	A	R	N	D	C	Q	E	G	H	I	L	K	M	F	P	S	T	W	Y	V
**CIDH920101**	-0,611	-0,406	-0,367	-1,654	0,598	-1,137	-0,952	-1,147	0,871	0,569	1,086	-0,523	1,164	1,271	-0,289	-1,127	-0,854	1,173	1,280	1,056
**CIDH920105**	-0,117	-0,548	-0,891	-1,155	0,617	-1,214	-1,253	-0,920	0,118	1,636	0,980	-0,538	0,843	1,185	-0,225	-1,086	-0,891	1,538	0,950	0,970
**ESID840101**	0,623	-2,534	-0,775	-0,901	0,293	-0,854	-0,744	0,481	-0,398	1,376	1,062	-1,497	0,638	1,188	0,120	-0,178	-0,053	0,811	0,261	1,078
**MANP780101**	0,060	-0,733	-0,924	-1,285	1,114	-0,708	-0,625	-0,282	-0,454	1,774	1,285	-0,962	0,962	0,714	-0,955	-1,044	-0,752	0,670	0,346	1,800
**PONP800101**	-0,177	-0,713	-1,045	-1,066	1,620	-0,855	-0,917	-0,360	0,202	1,511	1,057	-1,181	1,213	0,603	-0,917	-0,869	-0,605	0,277	0,508	1,715
**PONP800102**	-0,172	-0,688	-1,096	-1,090	1,938	-0,777	-0,962	-0,369	-0,025	1,339	0,944	-1,383	1,237	0,701	-0,796	-0,612	-0,516	0,332	0,408	1,587
**PONP800103**	-0,158	-0,667	-1,176	-1,119	1,905	-0,667	-1,063	-0,384	-0,328	1,113	0,831	-1,600	1,396	0,944	-0,639	-0,243	-0,384	0,463	0,294	1,481
**PONP800104**	0,699	-1,129	-0,388	-1,360	1,347	-1,113	-0,149	2,018	-0,889	1,455	0,977	-0,604	0,506	1,031	-0,951	-1,144	0,198	-0,527	-0,080	0,105
**PONP800105**	0,244	-0,802	-1,917	-0,387	1,244	-1,740	-0,533	-0,079	0,821	-0,140	1,698	-0,771	1,498	-0,079	-0,140	-0,710	0,167	-0,294	0,367	1,552
**PONP800106**	-0,821	-0,553	-0,694	-1,145	1,631	-0,088	-0,088	-0,624	0,165	0,785	0,870	-1,343	2,230	1,011	-0,856	-0,462	-0,920	-0,300	-0,222	1,426
**PRAM900101REVERSE**	0,607	-2,235	-0,701	-1,600	0,690	-0,560	-1,395	0,485	-0,335	0,915	0,851	-1,517	0,973	1,037	0,241	0,402	0,524	0,666	0,138	0,812
**SWER830101**	-0,401	-0,591	-0,921	-1,311	0,170	-0,911	-1,221	-0,671	-0,641	1,250	1,220	-0,671	1,020	1,920	-0,491	-0,551	-0,281	0,500	1,670	0,910
**NADH010101**	0,716	-1,875	-0,900	-0,943	1,337	-1,393	-1,307	-0,022	-0,686	1,241	1,112	-0,162	0,930	1,080	-0,751	-0,269	0,020	0,727	-0,022	1,166
**NADH010102**	0,590	-1,354	-0,756	-0,696	1,448	-1,195	-1,065	-0,048	-0,467	1,139	1,109	-1,963	0,810	1,159	-0,706	-0,178	0,052	0,770	0,191	1,159
**NADH010103**	0,357	-1,240	-0,867	-0,580	1,720	-1,187	-1,038	-0,271	-0,452	1,028	1,017	-1,655	0,741	1,284	-0,942	-0,409	0,027	1,007	0,304	1,156
**NADH010104**	0,196	-1,190	-0,949	-0,469	1,834	-1,190	-0,960	-0,393	-0,426	1,004	0,982	-1,506	0,742	1,288	-1,048	-0,524	0,065	1,135	0,327	1,080
**NADH010106**	-0,232	-0,946	-1,176	0,228	2,288	-1,061	-0,382	-0,831	-0,865	0,665	0,654	-0,727	0,665	1,057	-1,475	-0,762	0,619	1,206	0,021	1,057
**NADH010107**	-0,434	-0,754	-1,213	1,617	2,281	-0,721	0,542	-0,959	-0,992	-0,188	-0,122	0,526	0,091	0,567	-1,500	-0,844	1,010	1,051	-0,475	0,518
**WILM950101**	-0,390	-0,910	-0,281	-0,538	-0,143	-0,246	-0,481	-0,304	-1,706	1,568	1,579	-1,351	-0,304	2,324	-0,017	-0,779	-0,052	0,887	0,658	0,486
**WILM950102**	0,475	0,069	-0,686	-1,155	-0,089	-0,811	-0,441	-0,650	-0,528	1,001	1,655	-1,137	-1,238	2,422	-0,343	-0,722	0,233	1,457	0,108	0,380
**WILM950103REVERSE**	1,008	1,541	0,206	0,248	-2,545	0,488	0,092	1,076	-1,853	-0,505	0,206	1,242	-0,908	0,917	-0,538	-0,041	-0,275	-0,372	-0,294	0,307
**WILM950104**	-1,374	0,196	0,678	-0,633	1,563	-0,378	0,077	-0,864	-1,637	2,452	-0,007	0,180	-0,194	0,583	-0,501	0,328	-0,366	0,989	-1,470	0,379
**KUHL950101REVERSE**	0,350	-2,003	-0,885	-1,326	1,026	-0,856	-1,621	0,644	-0,268	1,262	0,997	-0,591	0,703	1,262	0,615	-0,297	-0,444	0,585	-0,297	1,144
**JURD980101**	0,547	-1,503	-0,974	-1,007	1,010	-1,033	-0,874	-0,028	-0,874	1,671	1,439	-1,175	0,811	1,109	-0,445	0,018	-0,048	0,031	-0,246	1,572
**KIDA850101REVERSE**	0,263	-1,822	-0,789	-0,789	1,023	-1,072	-1,140	0,156	-0,273	0,750	1,072	-1,656	0,711	1,393	0,731	-0,409	-0,614	1,530	0,546	0,390
**ENGD860101REVERSE**	0,607	-2,234	-0,701	-1,600	0,689	-0,558	-1,396	0,484	-0,333	0,914	0,852	-1,519	0,975	1,036	0,239	0,403	0,525	0,668	0,137	0,811
**KYTJ820101**	0,767	-1,342	-1,008	-1,008	1,001	-1,008	-1,008	0,030	-0,907	1,671	1,436	-1,142	0,800	1,101	-0,372	-0,104	-0,070	-0,137	-0,271	1,570
**ENGEL**	0,607	-2,234	-0,701	-1,600	0,689	-0,558	-1,396	0,484	-0,333	0,914	0,852	-1,519	0,975	1,036	0,239	0,403	0,525	0,668	0,137	0,811
**JONES**	-0,468	-0,485	-1,131	-0,647	0,087	-1,209	-0,637	-1,124	-0,468	1,478	0,642	0,189	0,216	1,238	1,153	-1,148	-1,148	2,006	1,069	0,385
**CIDBB**	-0,358	-0,368	-0,963	-0,973	0,462	-0,671	-1,559	-1,100	0,140	1,077	0,930	-0,368	0,960	1,213	-0,290	-1,188	-0,856	1,916	1,213	0,784
**CIDA**	0,233	-0,623	-0,993	-1,178	0,564	-1,139	-0,925	-0,915	-0,273	1,994	1,031	-0,662	1,060	0,865	-0,176	-0,701	-1,285	1,157	0,904	1,060
**CIDAB**	0,090	-0,759	-0,954	-1,100	1,134	-1,247	-1,237	-0,632	-0,320	1,934	0,860	-0,681	0,509	1,182	-0,281	-0,886	-0,681	1,397	0,568	1,104
**PONG1**	0,204	-0,283	-0,792	-1,257	1,141	-0,747	-1,025	-0,433	-0,268	1,920	1,058	-1,324	0,631	0,833	-1,287	-0,822	-0,493	0,886	0,594	1,463
**PONG2**	0,376	-0,253	0,124	-1,498	1,695	-0,527	-2,068	0,243	0,791	0,799	-0,061	-1,476	-0,039	-0,135	-0,379	0,710	-0,646	2,117	0,036	0,191
**PONG3**	0,321	-0,300	-0,366	-1,534	1,580	-0,706	-1,724	-0,101	0,297	1,506	0,553	-1,559	0,330	0,388	-0,921	-0,060	-0,631	1,671	0,346	0,909
**KIDER**	0,263	-1,822	-0,789	-0,789	1,023	-1,072	-1,140	0,156	-0,273	0,750	1,072	-1,656	0,711	1,393	0,731	-0,409	-0,614	1,530	0,546	0,390
**WOLR790101**	1,119	-2,548	-0,830	-0,830	0,589	-0,780	-0,920	1,198	-0,930	1,159	1,178	-0,800	0,549	0,669	0,539	-0,050	-0,020	-0,190	-0,230	1,129
**CHOTA**	-1,264	1,253	-0,235	-0,463	-0,807	0,223	0,452	-2,180	0,566	0,109	-0,006	0,681	0,338	0,910	-0,578	-1,264	-0,692	1,940	1,367	-0,349
**ROSEB**	-1,107	0,683	-0,726	-0,845	-0,033	-0,344	-0,463	-1,680	0,539	0,587	0,730	-0,415	0,945	1,446	-0,964	-1,131	-0,654	2,186	1,064	0,181
**ROSEAREVERSE**	1,385	-1,608	0,344	0,496	0,777	-0,242	-0,090	2,035	-0,437	0,018	-0,242	-0,957	-0,459	-0,892	0,756	1,124	0,647	-1,825	-1,196	0,365
**COHEN**	-0,874	-0,665	-1,607	-1,189	-0,456	-0,874	-0,665	-1,189	0,382	0,696	0,801	0,487	1,534	1,220	1,115	-0,979	-0,246	1,744	0,487	0,278
**JACWH**	0,626	-3,164	-0,379	-1,099	0,687	-0,325	-0,922	0,565	-0,501	0,755	0,782	-1,214	0,803	0,844	0,368	0,232	0,273	0,497	0,450	0,721
**CASSI**	0,198	-0,671	-0,478	-1,347	1,839	-1,057	-1,250	-0,092	0,391	1,356	0,487	-1,540	0,487	0,970	-0,960	-0,671	-0,381	1,549	0,487	0,681
**MDK0**	0,673	-1,499	-0,973	-1,006	1,002	-1,032	-0,874	-0,097	-0,874	1,660	1,430	-1,173	0,804	1,101	-0,446	0,015	-0,051	0,028	-0,249	1,561
**MDK1**	0,746	-1,581	-0,949	-0,949	0,846	-1,248	-1,049	0,081	-0,750	1,710	1,378	-1,182	0,846	1,046	-0,317	-0,052	0,115	0,115	-0,218	1,411

**BULDG**	0,737	0,823	1,037	0,737	0,468	1,123	0,629	0,951	0,823	-1,475	-1,690	0,576	-0,627	-1,551	-0,101	0,533	0,393	-1,207	-1,454	-0,724
**GUYH850101**	0,175	1,832	0,523	0,798	-1,216	0,953	0,844	0,386	-0,374	-0,951	-0,996	1,365	-1,372	-1,857	0,752	0,560	0,148	-0,383	-0,108	-1,079
**MIYS850101**	-0,166	-0,704	-0,923	-0,979	1,050	-0,849	-0,951	-0,563	-0,274	1,470	1,302	-1,279	1,527	1,564	-0,849	-0,746	-0,559	0,923	0,096	0,909
**WILSON**	-0,631	-0,848	-0,604	-0,929	1,157	-0,604	-0,550	-0,225	-0,902	0,615	1,238	-1,525	0,127	1,482	0,046	-0,712	-1,146	1,590	1,373	1,048
**CHOC760103**	0,586	-1,490	-0,873	-0,704	1,260	-1,153	-0,536	0,474	-0,592	1,821	0,979	-1,378	0,699	1,260	-0,536	-0,311	-0,255	-0,031	-0,704	1,484
**EISEN**	0,620	-2,531	-0,780	-0,900	0,290	-0,850	-0,740	0,480	-0,400	1,380	1,060	-1,500	0,640	1,190	0,120	-0,180	-0,050	0,810	0,260	1,080
**JANJ790102**	0,589	-1,824	-0,547	-0,689	1,441	-0,831	-0,831	0,589	0,021	1,157	0,873	-2,392	0,731	0,873	-0,263	0,021	-0,121	0,589	-0,405	1,015
**RAOARGOS**	0,954	-1,390	-1,041	-1,467	0,780	-1,041	-1,196	0,431	-0,363	1,109	1,167	-1,506	1,070	1,361	-0,634	0,199	0,412	0,257	-0,073	0,973
**NOZY710101TANFORD**	0,491	-2,485	-0,832	-0,180	0,180	-0,898	-0,794	0,359	-0,472	1,209	1,351	-1,512	0,510	1,030	0,019	-0,265	-0,142	0,671	0,151	1,606
**WELLING**	0,946	0,585	-0,270	0,629	-0,542	0,148	-0,232	-0,947	2,193	-1,631	0,693	1,522	-2,219	-0,675	-0,118	0,053	-0,067	-0,504	0,300	0,135
**PARJ860101**	0,177	0,508	0,951	1,424	0,066	0,793	1,077	0,745	0,177	-1,418	-1,608	0,745	-0,818	-1,608	0,177	0,872	0,666	-1,734	-0,455	-0,739
**ROSG850102**	0,030	-0,837	-0,924	-1,010	1,505	-1,010	-1,010	-0,143	0,377	1,244	0,984	-1,877	0,984	1,244	-0,837	-0,663	-0,317	0,984	0,204	1,071
**BISHOP**	0,200	0,321	0,442	1,411	-0,406	0,684	2,381	-0,042	0,200	-0,406	-0,769	0,563	-0,285	-1,375	0,442	0,079	0,079	-2,344	-1,254	0,079
**WIMLEYREVERSE**	-0,372	-1,499	-0,673	-0,312	0,075	-0,604	-0,037	-0,931	-0,037	1,021	1,133	-2,350	0,634	1,529	-0,062	-0,338	-0,157	1,855	0,669	0,454
**WIMLEY**	-0,086	-0,832	-0,377	-1,322	0,393	-0,564	-2,244	0,101	-0,086	0,474	0,766	-1,042	0,381	1,431	-0,412	-0,039	-0,051	2,271	1,209	0,031
**ARGP820101**	-0,469	-0,481	-1,135	-0,651	0,088	-1,208	-0,639	-1,123	-0,469	1,480	0,645	0,185	0,221	1,238	1,153	-1,147	-1,147	2,001	1,068	0,390
**FAUJ830101**	-0,167	-1,449	-1,051	-1,216	1,027	-0,682	-1,089	-0,468	-0,342	1,280	1,183	-1,429	0,726	1,270	0,231	-0,507	-0,216	1,717	0,464	0,717
**JOND750101**	-0,467	-0,484	-1,132	-0,646	0,087	-1,209	-0,637	-1,123	-0,467	1,477	0,642	0,190	0,215	1,239	1,153	-1,149	-1,149	2,006	1,068	0,386
**LEVM760101REVERSE**	0,089	-1,795	-0,288	-1,526	0,358	-0,288	-1,526	-0,180	0,089	0,789	0,789	-1,795	0,519	1,165	0,573	-0,342	0,035	1,650	1,058	0,627
**ZIMJ680101**	-0,427	-0,427	-1,137	-0,609	0,196	-1,223	-0,600	-1,127	-0,168	1,720	1,193	0,311	0,119	1,414	1,366	-1,089	-0,705	-0,926	1,625	0,493
**NADH010105**	0,012	-1,206	-1,171	-0,272	2,022	-1,301	-0,946	-0,603	-0,639	0,934	0,946	-0,378	0,792	1,277	-1,277	-0,698	0,130	1,100	0,237	1,041
**PONP930101**	0,204	-0,283	-0,792	-1,257	1,141	-0,747	-1,025	-0,433	-0,268	1,920	1,058	-1,324	0,631	0,833	-1,287	-0,822	-0,493	0,886	0,594	1,463
**COWR900101**	0,238	-1,310	-0,896	-0,489	0,566	-0,841	-0,380	-0,091	-1,873	1,324	1,317	-1,678	0,832	1,270	0,582	-0,591	-0,294	1,051	0,308	0,957
**BLAS910101**	0,276	-1,563	-0,851	-1,474	0,454	-0,821	-1,444	-0,080	-1,059	1,225	1,225	-0,732	0,632	1,403	0,543	-0,495	-0,228	1,047	1,047	0,898
**FASG890101**	0,232	1,030	0,634	0,772	-1,772	0,827	1,095	0,304	-0,118	-1,349	-1,304	1,638	-0,954	-1,294	0,562	0,903	0,573	-0,837	-0,043	-0,899
**FAUCH**	-0,168	-1,449	-1,050	-1,216	1,028	-0,683	-1,089	-0,468	-0,341	1,279	1,184	-1,428	0,725	1,268	0,231	-0,507	-0,214	1,717	0,464	0,718
**PONNU**	-0,176	-0,712	-1,046	-1,066	1,621	-0,855	-0,916	-0,360	0,202	1,512	1,058	-1,182	1,212	0,601	-0,916	-0,868	-0,606	0,277	0,506	1,713
**JANIN**	0,245	-1,091	-0,839	-0,839	2,661	-0,924	-0,924	0,327	-0,505	1,410	0,828	-1,132	0,408	0,661	-0,672	-0,505	-0,590	0,160	0,078	1,243
**GUYFE**	-0,219	-1,057	-0,670	-1,015	1,308	-0,940	-0,983	-0,595	0,371	1,255	1,145	-1,423	1,209	1,653	-0,908	-0,691	-0,446	0,790	0,200	1,017
**CHOTH**	0,585	-1,489	-0,873	-0,705	1,259	-1,154	-0,534	0,475	-0,591	1,822	0,981	-1,379	0,699	1,259	-0,534	-0,309	-0,256	-0,032	-0,705	1,483
**VHEG790101**	0,731	-2,592	-0,323	-1,555	-0,303	-0,187	-1,139	0,460	-0,455	1,137	1,104	-0,676	0,526	1,375	-0,425	0,050	0,219	1,002	0,050	1,002
**ROSEM**	0,224	-1,909	-1,151	-0,824	0,603	-1,081	-0,922	-0,090	-0,599	1,323	1,323	-1,240	0,692	1,323	0,729	-0,136	0,107	1,248	-0,548	0,930
**LEVIT**	0,089	-1,795	-0,288	-1,526	0,358	-0,288	-1,526	-0,181	0,089	0,789	0,789	-1,795	0,520	1,165	0,575	-0,343	0,033	1,651	1,058	0,627
**GIBRA**	-0,232	-1,030	-0,634	-0,772	1,772	-0,827	-1,095	-0,304	0,118	1,349	1,304	-1,638	0,954	1,294	-0,562	-0,903	-0,573	0,837	0,043	0,899
**ROSEF**	0,029	-0,837	-0,923	-1,010	1,503	-1,010	-1,010	-0,143	0,378	1,244	0,985	-1,879	0,985	1,244	-0,837	-0,664	-0,316	0,985	0,205	1,071
**SWEETEISENBERG**	-0,183	-1,822	-0,528	-0,800	1,191	-0,952	-0,843	-0,392	0,358	0,929	1,234	-1,360	1,294	1,825	-0,753	-0,564	-0,156	0,368	0,096	1,055
**NNEIG**	0,162	-0,284	-0,891	-1,448	1,854	-0,828	-1,383	0,057	-0,143	1,549	0,825	-1,501	0,582	0,733	-0,979	-0,586	-0,156	0,762	0,378	1,297
**SWEET**	-0,506	-1,768	-1,067	-0,424	0,143	-0,678	-0,443	-0,226	-0,232	1,341	1,341	-1,787	0,475	1,392	0,596	-0,761	-0,487	1,404	0,812	0,876
**WOLR810101REVERSE**	1,099	-2,502	-0,816	-1,025	0,575	-0,766	-0,901	1,173	-0,913	1,133	1,155	-0,789	0,535	0,654	0,779	-0,055	-0,025	-0,190	-0,227	1,107
**COWANWHITTACKER**	0,240	-1,196	-0,800	-1,701	0,558	-0,754	-1,546	-0,032	-0,536	1,389	1,366	-1,227	0,822	1,280	0,620	-0,521	-0,241	1,016	0,271	0,993
**ROSM880101**	-0,600	2,139	1,094	1,414	-0,529	0,914	1,120	-0,456	0,363	-1,104	-1,104	0,858	-0,735	-1,151	-0,832	0,477	0,372	-1,070	-0,246	-0,924
**ROSM880102**	-0,203	1,895	1,150	0,828	-0,815	1,081	0,924	0,105	0,607	-1,285	-1,285	1,237	-0,663	-1,386	-0,700	0,151	-0,088	-1,211	0,556	-0,898
**ROSM880103REVERSE**	0,641	0,947	-0,885	-0,580	0,336	-0,275	-2,107	1,863	-1,191	0,641	0,031	0,641	0,947	-0,275	-0,885	0,641	0,641	0,031	-1,801	0,641
**SET1**	0,662	-2,482	-0,530	-1,870	1,751	-0,452	-0,836	0,880	0,145	0,677	1,267	-0,327	0,400	-0,460	-0,460	0,334	0,271	-0,135	0,249	0,917
**SET2**	0,850	-1,912	-1,430	-0,956	0,830	-0,511	-1,306	0,682	-0,093	1,075	1,260	-1,121	0,959	-0,740	-0,740	0,666	0,220	0,509	0,541	1,216
**SET3**	0,977	-2,185	-0,429	-1,437	0,551	-0,472	-0,666	0,648	-0,193	1,082	1,369	-0,898	0,877	-1,123	-1,123	0,563	0,601	0,838	-0,015	1,035

Table S2: The amino acid hydrophobicity value of the 25 best individual synthesized scales, selected by feature selection in 1000-fold Monte-Carlo cross-validation. Scale notation $S_{x,y-z}$ shows from which number of subsets x the number x of scales was synthesized, where y is the repetition number (ranging from 1 to 20), defining a specific scale set. Within this scale set, z is the number of the classifier, ranging from 1 to x.

**SCALE-ID**/AMINO ACID	**MCC of Validation**	A	R	N	D	C	Q	E	G	H	I	L	K	M	F	P	S	T	W	Y	V
$S_{2,1-2}$	**0,84**	0,148	0,400	-0,104	-0,657	2,115	-0,091	-0,470	0,830	-2,660	-0,219	-0,155	0,232	-1,232	1,214	0,646	0,059	-0,772	1,143	-0,648	0,220
$S_{1,7-1}$	**0,82**	0,027	0,163	0,301	-0,158	0,279	0,043	-0,277	0,923	-1,923	-0,206	-0,218	0,095	-2,435	-0,883	0,680	-0,564	1,507	1,998	0,535	0,114
$S_{1,8-1}$	**0,81**	-0,281	0,113	-0,647	-0,565	-0,603	0,581	-0,331	0,512	-1,171	-0,447	0,046	0,244	-2,074	2,504	0,722	0,383	-0,305	1,877	-0,757	0,198
$S_{8,12-6}$	**0,81**	0,657	-1,397	-0,051	0,624	0,095	-0,197	-0,750	2,613	-0,261	-0,173	-0,812	0,600	-0,747	0,681	0,557	-0,095	-1,883	1,101	-1,122	0,560
$S_{1,5-1}$	**0,81**	-0,320	0,168	-0,668	-0,751	0,182	0,410	-0,345	0,422	-0,552	-0,321	0,090	0,383	-0,730	3,038	0,747	0,645	-1,853	0,604	-1,469	0,320
$S_{1,14-1}$	**0,81**	-0,074	0,191	0,470	-0,274	2,543	-0,129	-0,294	0,670	-1,939	-0,165	-0,405	0,066	-2,105	-0,553	0,634	-0,118	-0,070	1,573	-0,177	0,158
$S_{2,15-1}$	**0,80**	0,570	-0,443	-0,272	0,587	-1,147	-0,262	-0,712	0,545	-1,100	-0,113	-0,922	0,528	0,210	1,112	0,703	-0,595	-0,628	2,784	-1,624	0,780
$S_{6,19-2}$	**0,80**	0,967	-0,347	1,165	-0,541	-0,280	-0,287	-0,838	1,725	-2,662	-0,572	-0,457	1,007	0,200	0,327	0,105	0,004	-1,150	1,440	-0,253	0,447
$S_{1,12-1}$	**0,80**	-0,302	0,369	-0,503	-0,698	1,481	0,282	-0,481	-1,267	-1,397	-0,180	-0,063	0,081	-1,487	1,779	1,217	0,222	0,402	1,498	-1,516	0,564
$S_{2,8-2}$	**0,80**	0,408	0,488	0,493	-0,548	0,141	-0,442	-0,580	3,006	-1,825	-0,082	-0,055	-0,070	-0,574	-0,601	1,222	-0,595	-1,191	0,877	-0,476	0,404
$S_{3,18-3}$	**0,79**	0,300	-0,627	0,533	0,410	0,161	-0,091	-0,559	2,035	-1,790	-0,263	-1,767	0,785	-0,843	-0,813	1,442	-0,817	-0,271	1,449	0,306	0,423
$S_{4,13-2}$	**0,79**	1,336	0,377	0,103	-0,003	0,060	-1,088	-0,682	1,376	-1,229	-0,457	-0,560	-0,095	0,254	1,494	0,113	-0,983	-1,474	1,875	-1,308	0,891
$S_{2,20-1}$	**0,79**	0,370	0,169	-0,040	-0,880	2,095	-0,042	-0,449	1,274	-2,804	0,395	0,086	0,414	0,489	0,103	0,525	-0,295	-1,674	-0,183	0,007	0,440
$S_{1,9-1}$	**0,79**	0,201	0,189	0,479	0,046	-0,679	-0,093	-0,250	3,421	-1,334	-0,077	-0,140	0,124	-1,054	-1,011	0,401	-0,692	-0,939	0,759	0,633	0,018
$S_{2,17-1}$	**0,79**	1,038	-0,054	-0,341	-1,319	0,792	-0,521	-0,698	-0,974	0,076	0,037	-1,118	0,627	-0,903	-0,769	1,175	-0,637	-0,113	2,891	0,100	0,712
$S_{1,18-1}$	**0,79**	0,108	0,354	-0,524	-0,468	-0,488	0,286	-0,222	-0,249	-2,629	-0,295	0,284	-0,104	0,672	3,077	-0,034	0,209	-0,137	0,654	-0,647	0,154
$S_{1,13-1}$	**0,79**	0,001	-0,165	-0,556	-0,970	0,570	0,112	-0,548	-1,051	0,395	-0,234	-0,901	0,438	-2,447	0,197	1,376	-0,189	0,745	2,412	0,176	0,640
$S_{2,5-2}$	**0,78**	-0,266	-0,015	0,315	-0,009	1,949	0,221	-0,185	-0,571	-2,571	-0,234	-0,229	0,316	-1,906	0,156	0,320	0,088	1,037	1,709	-0,189	0,063
$S_{2,13-2}$	**0,78**	-0,365	-0,036	0,093	-1,141	0,104	0,476	-0,303	-1,614	-0,574	-0,438	-0,180	0,108	-2,218	1,182	0,474	0,249	1,151	2,437	0,380	0,214
$S_{7,5-1}$	**0,78**	1,121	0,704	1,564	-1,824	-1,278	-0,079	-1,114	1,141	-0,782	-0,716	-0,989	-0,736	0,297	0,832	-0,114	-0,053	-0,897	1,260	0,634	1,028
$S_{3,3-3}$	**0,78**	-0,281	0,201	0,217	-0,555	2,344	0,009	-0,318	-0,190	-0,891	-0,316	-0,478	0,018	-2,212	-0,294	0,980	0,385	-0,334	2,216	-0,766	0,267
$S_{3,15-2}$	**0,78**	-0,029	0,459	0,349	-0,160	-1,829	0,308	-0,221	2,455	-1,405	-0,324	-0,090	-0,197	-1,302	0,704	0,524	-0,515	-0,948	1,812	0,154	0,256
$S_{8,5-3}$	**0,78**	0,970	0,053	-0,031	-0,607	-0,003	-1,245	-0,529	1,478	0,793	-0,399	-0,676	-0,996	-0,856	-2,010	0,938	-0,880	0,625	1,863	0,688	0,821
$S_{3,5-1}$	**0,78**	0,156	0,154	0,447	-0,863	-0,083	-0,167	-0,319	-1,004	-0,892	-0,375	-0,431	-0,316	-0,948	-0,226	1,095	0,158	0,172	3,605	-0,543	0,380
$S_{3,5-3}$	**0,78**	0,542	0,497	0,761	-0,518	1,459	-0,154	-0,865	1,831	-2,116	0,126	-0,539	0,098	0,724	-0,287	0,775	-0,152	-1,958	0,033	-0,985	0,730
